# A chromosome 5q31.1 locus associates with tuberculin skin test reactivity in HIV-positive individuals from tuberculosis hyper-endemic regions in east Africa

**DOI:** 10.1371/journal.pgen.1006710

**Published:** 2017-06-19

**Authors:** Rafal S. Sobota, Catherine M. Stein, Nuri Kodaman, Isaac Maro, Wendy Wieland-Alter, Robert P. Igo, Albert Magohe, LaShaunda L. Malone, Keith Chervenak, Noemi B. Hall, Mecky Matee, Harriet Mayanja-Kizza, Moses Joloba, Jason H. Moore, William K. Scott, Timothy Lahey, W. Henry Boom, C. Fordham von Reyn, Scott M. Williams, Giorgio Sirugo

**Affiliations:** 1 Vanderbilt Genetics Institute, Vanderbilt University, Nashville, Tennessee, United States of America; 2 Geisel School of Medicine at Dartmouth, Hanover, New Hampshire, United States of America; 3 Department of Population and Quantitative Health Sciences, Case Western Reserve University, Cleveland, Ohio, United States of America; 4 Tuberculosis Research Unit, Case Western Reserve University, Cleveland, Ohio, United States of America; 5 Tokyo Medical and Dental University, Tokyo, Japan; 6 Muhimbili University of Health and Allied Sciences, Dar es Salaam, Tanzania; 7 Uganda-CWRU Research Collaboration, Kampala, Uganda; 8 College of Health Sciences Makerere University and Mulago Hospital, Kampala, Uganda; 9 Perelman School of Medicine at the University of Pennsylvania, Philadelphia, Pennsylvania, United States of America; 10 John P. Hussman Institute for Human Genomics, University of Miami, Miami, Florida, United States of America; 11 Centro di Ricerca, Ospedale San Pietro Fatebenefratelli, Rome, Italy; The University of Melbourne, AUSTRALIA

## Abstract

One in three people has been infected with *Mycobacterium tuberculosis* (MTB), and the risk for MTB infection in HIV-infected individuals is even higher. We hypothesized that HIV-positive individuals living in tuberculosis-endemic regions who do not get infected by *Mycobacterium tuberculosis* are genetically resistant. Using an “experiment of nature” design that proved successful in our previous work, we performed a genome-wide association study of tuberculin skin test positivity using 469 HIV-positive patients from prospective study cohorts of tuberculosis from Tanzania and Uganda to identify genetic loci associated with MTB infection in the context of HIV-infection. Among these individuals, 244 tested were tuberculin skin test (TST) positive either at enrollment or during the >8 year follow up, while 225 were not. We identified a genome-wide significant association between a dominant model of rs877356 and binary TST status in the combined cohort (Odds ratio = 0.2671, p = 1.22x10^-8^). Association was replicated with similar significance when examining TST induration as a continuous trait. The variant lies in the 5q31.1 region, 57kb downstream from *IL9*. Two-locus analyses of association of variants near rs877356 showed a haplotype comprised of rs877356 and an *IL9* missense variant, rs2069885, had the most significant association (p = 1.59x10^-12^). We also replicated previously linked loci on chromosomes 2, 5, and 11. IL9 is a cytokine produced by mast cells and T_H_2 cells during inflammatory responses, providing a possible link between airway inflammation and protection from MTB infection. Our results indicate that studying uninfected, HIV-positive participants with extensive exposure increases the power to detect associations in complex infectious disease.

## Author summary

Approximately one-third of the world’s population has been exposed to *Mycobacterium tuberculosis*, the bacterium that causes tuberculosis. A small number of those infected develop active disease; however, there is a substantial portion of exposed people who do not even show evidence of an immunological response. These people who appear to resist infection, as measured by a negative tuberculin skin test, represent a subpopulation from which we can learn about resistance. We used a genome-wide approach to study the genetic basis of this resistance in unique cohorts of hypervulnerable, HIV-positive individuals from Uganda and Tanzania, in which exposure was virtually assured. We identified one locus that was highly significantly associated and conferred more than 70% protection from infection. The most significant variant, rs8773656, was near *IL9* and *SLC25A48*, and a haplotype including this variant and a missense mutation in *IL9* was even more significantly associated with negative skin tests. Although it is impossible based solely on our data to determine the causal variant or genes, IL9 is an attractive candidate as its product has previously been associated with bronchial hyperresponsiveness, thereby providing a possible link between inflammation and protection from *Mycobacterium tuberculosis* infection.

## Introduction

One third of the world’s population has been infected with *Mycobacterium tuberculosis* (MTB)[1, 2]. Subsequent tuberculosis disease (TB) occurs during the lifespan of about 10% of those infected[1–3]. Tuberculosis is a major cause of morbidity and mortality worldwide, with 1.5 million deaths and 9.6 million new cases of active disease reported in 2014[1]. Tuberculosis is the primary cause of death in people co-infected with the human immunodeficiency virus (HIV), and 400,000 of the global TB deaths in 2014 occurred in this patient population [1, 4]. The immunosuppression from HIV facilitates progression to active disease directly following infection, or by the reactivation of a latent MTB infection[5, 6]. While the clinical trajectory of a given MTB infection has many determinants and possible outcomes, infection *per se* is a necessary prerequisite. Of note, about 10–20% of people living in areas hyperendemic for MTB, which virtually guarantees repeated exposure, appear to be resistant to infection[7–10].

Historically, MTB infection has been evaluated with a tuberculin skin test (TST) measuring the induration caused by a delayed type hypersensitivity reaction to an intradermal injection of MTB purified protein derivative (PPD)[11, 12]. In endemic areas, induration ≥ 5mm measured between 48 and 72 hours post-injection is indicative of infection. A study of TST reactivity among siblings demonstrated high heritability, suggesting a possible genetic component to the MTB infection resistance phenotype[13, 14]. Several studies have capitalized on this finding and identified loci relevant to the MTB infection phenotype. A family-based linkage analysis of TST response identified *SLC6A3* and a region on chromosome 11 (p14) as linked to infection[8]. A full genome microsatellite scan comparing persistent MTB negative patients to those with latent infections identified an association with the *SLC11A1* gene, and candidate regions on chromosomes 2 (q14, q21-q24) and 5 (p13-q22)[15].

Recently, novel methods for evaluating MTB infection status have been developed. Interferon-gamma release assays (IGRAs) detect the concentration of IFN-γ in response to a mixture of MTB-specific antigens[16, 17]. The purified protein derivative used in TST has some antigenic overlap with the Bacille Calmette-Guérin (BCG) vaccine, although 10 years post-vaccination the confounding effect is minor; approximately 1% of adult subjects inoculated at birth with BCG are TST-false positive [18]. IGRAs’ antigens have no overlap with the BCG vaccine, and maintain excellent specificity in individuals who had childhood BCG vaccinations [16, 17]. However, in people with compromised immune systems and previously exposed to MTB, anergy due to immunodeficiency may prevent detection of a positive TST and/or IGRAs. Inclusion of negative and positive assay controls allows us to better assess this potential confounder.

We used a genome-wide approach to evaluate common variants for association with TST response in a patient population that hypothetically allows us to identify extreme genetic effects. Namely, we hypothesized that HIV-positive individuals who live in areas endemic for tuberculosis but who do not get infected, are strongly genetically resistant to MTB. Using two recently concluded prospective cohorts of tuberculosis disease from Tanzania and Uganda, with available TST and IFN-γ results, we identified a variant on chromosome 5q31.1, near *SLC25A48* and *IL9* that imparts resistance to MTB infection in immunocompromised individuals.

## Results

Sex was significantly associated with TST status in the combined Ugandan and Tanzanian cohorts (Odds Ratio (OR) for males 1.91, 95% confidence interval (CI) 1.27–2.86, p = 0.002; Table 1), but it did not associate when studied in Uganda (p = 0.762; Table 2) or Tanzania (p = 0.349; Table 3) alone. Age was not significantly associated with TST status in the combined cohort (p = 0.108; Table 1), nor in Uganda (p = 0.384; Table 2) or Tanzania (p = 0.153; Table 3) alone. Therefore, all analyses below were adjusted for sex, 10 principal components, and cohort of origin when Tanzanian and Ugandan datasets were combined.

**Table 1:** Summary statistics of study participants in the combined Ugandan and Tanzanian cohorts. Odds ratio result is from univariate logistic regression.

Combined Data		TST^+^	TST^-^	Odds Ratio	95% Confidence Interval	p value
n		244	225			
Age (st. dev.)		32.43 (8.02)	34.49 (7.96)			0.108
Sex	Male	89	52			
	Female	155	173	1.91	(1.27, 2.86)	0.002
CD4* (st. dev)		430.17 (262.45)	430.12 (234.67)			0.99

*CD4 data available for 151 TST+ patients and 198 TST- patients in the combined cohort

**Table 2:** Summary statistics of study participants in the household contact study in Uganda. Odds ratio result is from univariate logistic regression.

Uganda HHC Data		TST^+^	TST^-^	Odds Ratio	95% Confidence Interval	p value
n		150	49			
Age (st. dev.)		32.72 (7.78)	31.57 (8.76)			0.384
Sex	Male	68	21			
	Female	82	28	1.11	(0.58, 2.12)	0.762
CD4* (st. dev; range)		504.76 (250.47; 15–906)	463.19 (215.82; 1–673)			0.16

*CD4 data available for 57 TST+ patients and 22 TST- patients in the Uganda cohort

**Table 3:** Summary statistics of study participants in the extended follow up of the DarDar vaccine trial in Tanzania. Odds ratio result is from univariate logistic regression.

Tanzania, DarDar Data Vaccine		TST^+^	TST^-^	Odds Ratio	95% Confidence Interval	p value
n		94	176			
Age (st. dev.)		33.50 (8.56)	34.95 (7.61)			0.153
Sex	Male	21	31			
	Female	73	145	1.35	(0.72, 2.50)	0.349
CD4* (st. dev; range)		307.18 (235.77; 204–1490)	165.56 (214.49; 204–1390)			0.024

*CD4 data available for all patients in the Tanzania cohort

In logistic regression analysis adjusted for covariates, we observed a genome-wide significant association between a dominant genetic effect of rs877356 on chromosome 5q31.1 and binary TST status in the combined cohort (OR = 0.27, 95% CI 0.17–0.42, p = 1.22x10^-8^, Table 4, S1–S3 Figs). The variant had consistent effects in Uganda (OR = 0.17, 95% CI 0.08–0.37, p = 9.18x10^-6^; Table 5; S4 Fig) and Tanzania (OR = 0.33, 95% CI 0.18–0.59, p = 1.81x10^-4^; Table 6, S5 Fig). Linear regression analyses of continuous size of TST induration under a dominant genetic model produced similar results (combined cohort beta = -4.14, 95% CI -5.55 to -2.74, p = 1.45x10^-8^; S1 Table). Variant rs877356 met the multiple testing-adjusted threshold for this study (3.08x10^-7^) and was nearly genome-wide significant in an additive model using binary TST status (OR = 0.33, 95% CI 0.222–0.493, p = 5.45x10^-8^; S2 Table), and continuous size of TST induration (combined cohort beta = -3.34, 95% CI -4.53 to -2.14, p = 6.95x10^-8^; S3 Table). This SNP was in Hardy Weinberg equilibrium in Tanzania (p = 0.68) and Uganda (p = 0.21). No other unimputed SNPs were significant at the multiple testing corrected threshold in any of the genetic models tested (Tables 4–6, S1–S5 Tables).

**Table 4:** Single nucleotide polymorphisms associating with dichotomous tuberculin skin test status below a 5x10^-5^ p-value using a dominant genetic model in the combined cohort* (n = 469).

SNP	Chr.	Minor Allele	MAF	Odds Ratio	95% Confidence Interval	p value	Nearest gene
rs877356	5	T	0.2292	0.267	(0.170, 0.422)	1.22E-08	*SLC25A48/IL9*
rs7808481	7	A	0.2164	2.523	(1.630, 3.910)	3.33E-05	*Loc340268*
rs1880386	10	A	0.2132	2.462	(1.594, 3.801)	4.85E-05	*GRID1*

* adjusted for 10 principal components, sex, and cohort of origin

**Table 5:** Single nucleotide polymorphisms associating with dichotomous tuberculin skin test status below a 5x10^-5^ p-value using a dominant genetic model in the Ugandan cohort* (n = 199).

SNP	Chr.	Minor Allele	MAF	Odds Ratio	95% Confidence Interval	p value	Nearest gene
rs877356	5	T	0.2337	0.171	(0.078, 0.373)	9.18E-06	*SLC25A48/IL9*
rs654718	11	G	0.2136	0.190	(0.089, 0.406)	1.81E-05	*MRE11A*
rs7944514	11	C	0.4121	5.284	(2.457, 11.360)	2.03E-05	*POLD3*
rs7837658	8	T	0.4472	4.842	(2.319, 10.110)	2.67E-05	*RNF19A*

* adjusted for 10 principal components and sex

**Table 6:** Single nucleotide polymorphisms associating with dichotomous tuberculin skin test status below a 5x10^-5^ p-value using a dominant genetic model in the Tanzanian cohort* (n = 270).

SNP	Chr.	Minor Allele	MAF	Odds Ratio	95% Confidence Interval	p value	Nearest gene
rs17062122	6	C	0.3259	0.280	(0.161, 0.487)	6.20E-06	*Loc285735*
rs8142256	22	C	0.35	0.312	(0.181, 0.539)	2.87E-05	*FAM19A5*
rs10998959	10	T	0.2537	0.306	(0.173, 0.540)	4.33E-05	*Loc100129281*
rs11736841	4	T	0.2556	3.091	(1.792, 5.332)	4.96E-05	*ODZ3*
…	…	…	…	…	…	…	*…*
rs877356	5	T	0.2259	0.330	(0.184, 0.589)	1.81E-04	*SLC25A48/IL9*

* adjusted for 10 principal components and sex

**Fig 1:**
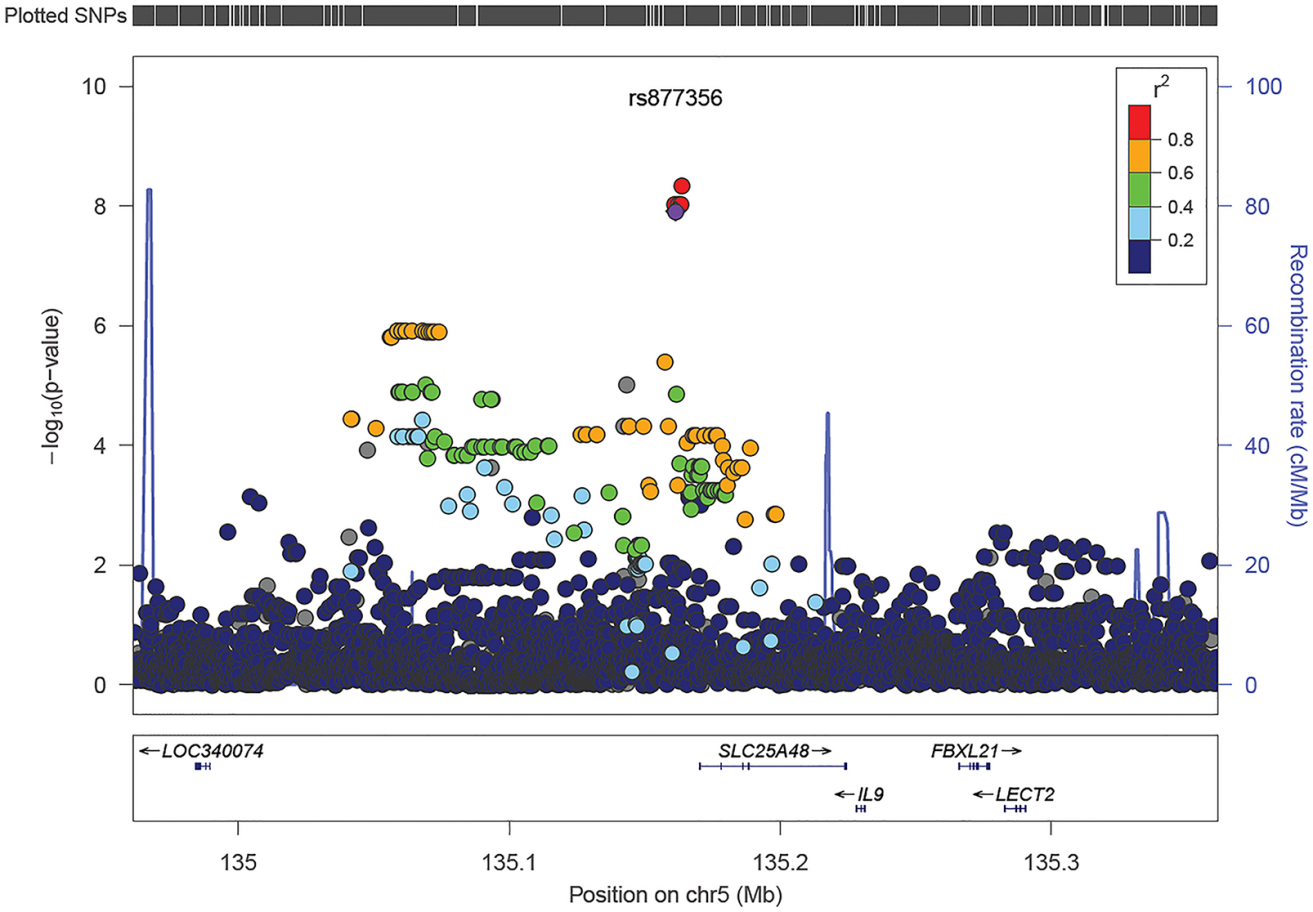
Locus zoom plot of results from a logistic regression association of dichotomous tuberculin skin test status with a dominant genetic model of imputed SNPs in the *SLC25A48/IL9* region in the combined cohort, adjusted for 10 principal components, sex, and cohort of origin; SNP with the most significant association of a genotyped SNP in the Exome Beadchip analysis in purple.

To evaluate SNPs in the region not included on our genotyping array, we imputed SNPs within 0.5 megabases of rs877356. One SNP, rs17169187, in high linkage disequilibrium (LD) with rs877356 (D’ = 1 in both cohorts, r^2^ = 0.99 in Tanzania, 0.98 in Uganda) and 2,340 bases away, is the variant with the most significant association to binary TST status using a dominant model (combined cohort OR = 0.26, 95% CI 0.16–0.40, p = 4.57x10^-9^; Fig 1, S6A Table). The results were consistent with those from linear regression on continuous size of TST induration (combined cohort beta = -4.29, 95% CI -5.69 to -2.88, p = 4.58x10^-9^; S7A Table). This variant is also genome-wide significant in additive modeling of both a binary TST designation (combined cohort OR = 0.320, 95% CI 0.214–0.478, p = 2.56x10^-8^; Fig 1, S6B Table) and continuous size of TST induration (combined cohort beta = -3.43, 95% CI -4.62 to -2.24, p = 2.84x10^-8^; S7B Table). Adjustment for CD4 count did not significantly affect our results (S8 Table).

In the Tanzanian cohort, IFN-γ responses to positive control (PHA) and negative control (medium) antigens did not differ by TST results, but were significantly higher in TST cases for all mycobacterial antigens (S9A Table). In Uganda, we observed the same trends; however, due to smaller sample sizes, the comparisons were not statistically significant (S9B Table). Separately, we examined the prevalence of TST-positivity in the entire Ugandan household contact study cohort, and found that although the prevalence of TST^+^ in HIV^+^ is significantly lower, it is still very high (71% in HIV^-^ versus 62% in HIV^+^, p = 0.003; S10 Table). Furthermore, the distribution of TST induration examined as a continuous variable did not differ by HIV status (p = 0.06). In the GWAS analysis, removing either potentially false negative subjects (n = 16), potentially false positive subjects (n = 20), or both did not affect the results substantially (S11–S14 Tables). The variant was also genome-wide significant when we included patients with prior tuberculosis in the analyses (S15 Table).

We found the strongest single variant association using a dominant model of rs877356; therefore, we used dominant coding of the SNP in 2-variant haplotype in the *SLC25A48* region while using additive models of all other SNPs. An rs877356-rs2069885 haplotype had the strongest association in this analysis (omnibus p = 1.59x10^-12^ in the combined cohort; Table 7). The haplotypes had similar association in the Ugandan (p = 2.51x10^-8^; Table 8) and Tanzanian cohorts (p = 1.37x10^-11^; Table 9), with the T-G haplotype frequencies being 0.32/0.60 and 0.20/0.45 in TST^+^/TST^-^ subjects, representing a similar enrichment in both cohorts (Tables 8 and 9). The haplotype, C-G, also had a consistent distribution between the cohorts, with a TST^+^/TST^-^ frequency of 0.58/0.33 in Uganda and 0.68/0.48 in Tanzania (Tables 8 and 9). The results were consistent in additive modeling of both SNPs (p = 2.59x10^-9^ in the combined cohort; S16 Table). The haplotype had similar association in the Ugandan (p = 1.03x10^-5^; S16B Table) and Tanzanian cohorts (p = 6.35x10^-5^; S16C Table). In addition, patterns of linkage disequilibrium (LD) were strikingly similar across the whole region in both Ugandan and Tanzanian cohorts (S6 and S7 Figs), an unexpected result given the greater variation (and reduced extent) of LD among African populations. Remarkably, in the same cohorts, high similarity in LD structure was previously found near *IL12B*, encompassing a variant associated with resistance to active TB in HIV^+^ individuals and displaying signals of strong selection[19].

**Table 7:** Association of the 2-variant haplotype using a dominant model of rs877356 with an additive model of rs2069885 with dichotomous TST induration status in the *SLC25A48/IL9* region in the combined cohort.

Haplotype	TST^+^	TST^-^	TST^+^ Freq	TST^-^ Freq
C-A	33	12	0.0679	0.02667
C-G	301	202	0.6193	0.4489
T-A	17	17	0.0350	0.0378
T-G	135	219	0.2778	0.4867
Likelihood ratio χ^2^ = 57.97 df = 3 p-value = 1.59E-12*				

* adjusted for principal components, sex, and cohort of origin

**Table 8:** Association of the 2-variant haplotype using a dominant model of rs877356 with an additive model of rs2069885 with dichotomous TST induration status in the *SLC25A48/IL9* region in the Ugandan cohort.

Haplotype	TST^+^	TST^-^	TST^+^ Freq	TST^-^ Freq
C-A	19	2	0.0633	0.0204
C-G	175	32	0.5833	0.3265
T-A	9	5	0.0300	0.0510
T-G	97	59	0.3233	0.6020
Likelihood ratio χ^2^ = 38.25 df = 3 p-value = 2.51E-08*				

* adjusted for principal components and sex

**Table 9:** Association of the 2-variant haplotype using a dominant model of rs877356 with an additive model of rs2069885 with dichotomous TST induration status in the *SLC25A48/IL9* region in the Tanzanian cohort.

Haplotype	TST^+^	TST^-^	TST^+^ Freq	TST^-^ Freq
C-A	14	10	0.0753	0.0284
C-G	126	170	0.6774	0.4830
T-A	8	12	0.0430	0.0341
T-G	38	160	0.2043	0.4545
Likelihood ratio χ^2^ = 53.59 df = 3 p-value = 1.37E-11*				

* adjusted for principal components and sex

We also determined whether previously associated or linked loci were significant in our results. Several regions previously shown to be linked to TST response were nominally significant in our study (10^−3^ > p > 10^−4^), including ones on chromosomes 2, 5 and 11 (S17 Table)[8, 15, 20]. Chromosome 11p14-15 associated with TST response in our analyses as it did previously[21]. Although our most significant region on chromosome 11 was distal to the linkage peak, the region directly under the peak was almost as significant (p~10^−3^) (S18 Table). Another previous association signal, IL-10, did not show signs of replication in our study (S17F Table). These results overall support the validity of our study design as most previous regions replicated.

## Discussion

In this study we examined the association of common genetic variants with *Mycobacterium tuberculosis* infection in HIV^+^ patients from the extended follow-up of the DarDar vaccine trial in Tanzania and the Household Contact study in Uganda. By applying the “experiment of nature” strategy outlined in a genetic study of tuberculosis disease with the same cohorts [19], we hypothesized that these immunosuppressed patients who live in MTB endemic areas but do not get infected have strong innate resistance. This hypothesis and approach were validated as we identified a novel association between protection from MTB infection and rs877356 with a large effect size. This variant is 9,119 bases upstream of the coding region of *SLC25A48[22]*, a *Homo sapiens* solute carrier family 25, member 48. *SLC25A48* is a mitochondrial carrier of amino acids[23, 24]. This SNP is also 57,662 bases downstream from *IL9*, which we think is a particularly compelling candidate.

Both genes have supporting evidence that may implicate them. With respect to *SLC25A48*, there is evidence from GTEX that this SNP is an eQTL for a lncRNA closer to it than *IL9* (http://gtexportal.org/home/eqtls/bySnp?snpId=rs877356&tissueName=All). In contrast, the involvement of *IL9* as the potentially causal gene in our association study was supported by our haplotype analyses. The rs877356-rs2069885 haplotype had the most significant association in this region. The SNP, rs2069885, is 66kb away from rs877356, and is a missense variant in *IL9* (Threonine (A**C**G) ->Methionine (A**T**G))[22]. While rs2069885 was not significant in univariate analyses (p = 0.091 in the combined cohort for TST as a binary outcome and with an additive model), the association of the haplotype was several orders of magnitude more significant than that of rs877356 alone.

Although we cannot at the present distinguish which of these two genes, if either, is the truly associating one, *IL9* is an attractive candidate for resistance to MTB infection because of its association with bronchial hyperresponsiveness [25], which is hereditary and a risk factor for asthma[25–27]. Of note, the prevalence of asthma in East Africa is high, especially in urban settings[28], childhood MTB infection protects from asthma, and an inverse relationship between incidence of active TB and asthma has been reported [29, 30]. IL9 was originally described as a T cell and mast cell growth factor, but has since been found to have pleiotropic effects on the immune system[31–33]. IL9 promotes IL4-mediated production of IgE and IgG antibodies[34, 35], and bronchial hyper-responsiveness is associated with elevated serum IgE levels[25, 36]. IL9 also promotes proliferation of hematopoietic progenitor cells[37, 38], and it has specific effects on lungs. In airway smooth muscle cells, IL9 induces the expression of chemokine CCL11, thereby inducing eosinophil chemotaxis and allergic reactions, and in airway epithelial cells, IL9 directly induces mucous production and stimulates IL13, which leads to further airway inflammation and perhaps reduced risk of MTB infection [31, 32, 39–41].

The TST phenotype can be studied both as a binary variable, < versus ≥ 5mm induration, or as a continuous outcome. Our single-SNP association results were consistent using both outcomes. Variant rs877356 was genome-wide significant in both logistic and linear regression models in the combined cohort using a dominant genetic model as well as at a multiple testing corrected level in an additive model. The most significant imputed variant in the region, rs17169187, was genome-wide significant for both outcomes in additive and dominant modeling.

One possible limiting factor of these conclusions is immune anergy, which is a potential confounder in studies of TST reactivity, especially in an HIV^+^ context. TST responses can be < 5mm because a patient has not been infected with MTB, or in case of anergy, is unable to mount a hypersensitivity reaction to PPD even if infected. However, we believe our results are unlikely to be confounded by anergy for several reasons. First, if anergy existed, it would result in misclassifying cases as controls, which would decrease power and underestimate effect sizes. Since we observed significant effects, this was not the case in our data. Second, we leveraged existing interferon-γ response data in both cohorts to evaluate confounding by immunosuppression. We removed all patients suspected of immune anergy prior to analysis, and further adjustment for a missing response variable did not affect the association of our variant, demonstrating the robustness of our findings. Particularly in the Tanzania data, where the reported rate of TST-positivity in HIV-infected is lower than in HIV-uninfected individuals[42], the PHA responses were quite high and did not differ by TST status, demonstrating that individuals do indeed mount immune responses. Analyses utilizing these immunologic data, where available, showed significant effects for the same SNP, suggesting our results are robust to immunological differences between subjects. Third, data from the entire household contact study in Uganda indicates that anergy is not an issue in that cohort: the prevalence of TST-positivity in HIV-infected individuals is 62%, compared to 71% in HIV-uninfected individuals and ~34% in HIV-uninfected community controls (S10 Table and [43]). This high rate of TST-positivity in the HIV-infected subjects is inconsistent with anergy being a major confounder in this population. Furthermore, since we see similar genetic effects in the Ugandan and Tanzanian cohort, it is unlikely that anergy is a problem in Tanzania and not in Uganda. Lastly, we replicated loci that had been previously associated with TST in independent HIV^-^ cohorts, further validating our design. Unfortunately, data on PHA and CD4+ count were unavailable for some of the subjects in this study, so we were unable to fully explore some of these potential explanations. In summary, the aforementioned sensitivity analyses and other factors make anergy an unlikely cause of the observed association in these data, though we cannot absolutely exclude this possibility. Future studies should examine this locus as a candidate for association with TST.

As we have previously shown for tuberculosis disease[19], the present study confirms that the choice of an extreme phenotype, HIV^+^ patients who live in MTB endemic areas but do not get infected, enriches for major, homogeneous genetic effects. This design permits the use of relatively small sample size even in a genome-wide association study. Although the small sample size is the biggest weakness in this study, the large and replicated effect size observed in this unique study design and populations allowed us to find significant associations in an apparently relevant region of the genome. The variant with the most significant association is near *IL9*, a gene with a substantial role in airway inflammation, bronchial asthma, and other respiratory infections[44, 45]. This, along with observational studies of the inverse incidence of asthma and tuberculosis, leads to the conclusion that the same gene whose over-expression plays a significant role in the pathogenesis of asthma, could also prevent MTB infection by the same mechanism.

## Material and methods

### Study populations

HIV^+^ subjects from a cohort in Tanzania and one in Uganda were included in this study. A complete description of the study cohorts and genetic analysis methods is provided in our previous work [19].

#### Tanzania

Patients from the extended follow-up cohort of the DarDar vaccine trial in Dar es Salaam, Tanzania were recruited for this study. The full cohort has been described elsewhere[46]. Briefly, the DarDar trial was a phase III randomized trial of SRL 172, an inactivated whole cell mycobacterial vaccine booster to a childhood Bacille Calmette-Guérin (BCG) vaccination. Subjects were enrolled between 2001 and 2005. Follow-up continued until the study was concluded in 2008. Recruited patients were HIV^+^-positive adults (≥18 years old) with a BCG scar, a CD4 count >200 cells/μl and were TB-negative at the time of enrollment. TST reactivity was measured at enrollment, preventing any confounding by the effects of the vaccine. A saline placebo was administered to 1007 patients, while 1006 patients received 5 doses of the vaccine. A routine follow-up for active TB (physical examination, chest radiograph, sputum samples for culture and acid fast bacilli (AFB) stain, and phlebotomy for an automated mycobacterial blood culture) was performed every 3 months for the duration of the study. Upon conclusion of the trial, an extended follow-up cohort of 800 participants from both the placebo and vaccine arm was selected for annual evaluation for active TB. Between September and December of 2013, 304 patients from the extended follow-up were recruited during their routine visits.

#### Uganda

We obtained 263 samples from HIV-positive participants from the Household Contact Study (HHC), conducted in Kampala, Uganda. This cohort has been previously described in detail[47, 48]. Briefly, the Uganda National Tuberculosis and Leprosy Programme referred patients diagnosed with new active tuberculosis to the study, and patients who consented were enrolled as index cases. Household contacts were defined as individuals living in the same household as the index case for at least 7 consecutive days in the 3 month period leading up to the diagnosis of the index case[10]. Household contacts were subsequently enrolled and evaluated for active TB, latent TB, and HIV. Recommended therapy was administered to all cases of active TB[49]. In contrast to the cohort from Tanzania, CD4 counts were not available until 2004 when antiretroviral drugs became available in Uganda. In subjects enrolled prior to 2004, antiretrovirals were not given to HIV-positive subjects. In subjects enrolled when antiretrovirals were available in Uganda, these drugs were administered after the diagnosis of HIV, which occurred after basic clinical data, including TST, and blood samples were obtained. Of note, the HHC study design guarantees exposure of the controls to MTB during the follow up[47]. We only analyzed adult participants (≥18 years old) of the HHC.

### Immune assays

#### Tanzania

Intradermal injections of purified protein derivative (0.1 ml, RT-23, Staten Serum Institute, Copenhagen) on the forearm were administered to all enrolled patients prior to vaccination, and resultant skin induration size was measured by trained personnel after 48–72 hours. Preventative isoniazid treatment (300 mg daily for 6 months) was offered to subjects with a positive TST using the criterion for HIV^+^ patients (≥5 mm as positive) as recommended by a consensus statement [50].

Immune response to *Mycobacterial* antigens was assessed with an interferon gamma (IFN-γ) enzyme linked immunosorbent assay (ELISA), a tritiated thymidine lymphocyte proliferation assay (LPA) and an ELISA for antibodies to the glycolipid lipoarabinomannan of MTB (LAM). The assays used in this study have been described in detail elsewhere[51]. Briefly, phlebotomy was performed prior to vaccination and at the conclusion of the study, and peripheral blood mononuclear cells (PBMCs) were isolated by ficoll density gradient centrifugation for IFN-γ and LPA assays, performed on site. Centrifuged, frozen serum was sent to Dartmouth College for LAM assays.

IFN-γ and LPA assays used four different antigens: *Mycobacterium Vaccae* sonicate (2 mcg/ml), *MTB* Antigen 85 (Ag85; 1 mcg/ml), *MTB* early secretory antigenic target 6 (ESAT-6; 2 mcg/ml), and *MTB* whole cell lysate (WCL; 1 mcg/ml)[51]. Importantly, ESAT-6 is not present in the childhood Bacille Calmette-Guérin (BCG) vaccine that is commonplace in East Africa; therefore, confounding by BCG status can be controlled with this additional data. Media alone was used as a negative control and phytohemagglutinin (PHA, 2.5 mcg/mL; Sigma, St. Louis, MO) was used as a positive control[51].

#### Uganda

Intradermal injections of purified protein derivative (5 tuberculin units) on the forearm were administered to study participants at enrollment, and 3, 6, 12, and 24 months post-enrollment, if the tests were negative at earlier time points[10]; PPD was also obtained from Serum Staten Institute as in Tanzania. The size of skin induration was measured by trained personnel 48–72 hours after each injection. For patients measured at multiple time points, the largest TST reaction was used in the analysis, and a 5 mm cutoff was used to define TST^+^, as recommended by a consensus statement[50]. In this phase of the HHC study, daily isoniazid preventative treatment was offered to all HIV^+^ subjects for 6–9 months [10, 15].

Briefly, phlebotomy was performed at enrollment. Whole blood was stimulated with MTB antigens: MTB culture filtrate CXFT, ESAT-6, and CFP10[10, 52], and the IFN-γ response was measured by ELISA (Thermo Scientific, Rockford, IL). Whole blood cultured without antigen stimulation served as a negative control. Phytohemagglutinin (PHA; Sigma, St. Louis, MO) was used as a positive control, while the IFN-γ response to media was subtracted from antigen-stimulated readings[10, 52]. Negative differences were considered a 0.

### DNA isolation and genotyping

For participants from the extended follow-up of the DarDar vaccine trial, 5ml of whole blood was drawn upon enrollment, and DNA was extracted the day of the phlebotomy using the Gentra Puregene Blood kit (QIAGEN) in accordance with the manufacturer’s recommendations. For participants of the Household Contact Study, buffy coats were isolated on site and shipped to Dartmouth College for DNA extraction. The QIAamp DNA Blood Mini Kit (QIAGEN) was used to isolate DNA from the buffy coats. DNA samples were stored at -80°C before genotyping. DNA quality was evaluated with the 260/280 ratio using a NanoDrop 2000 spectrophotometer at Dartmouth College (Thermo Scientific) and an Electrophoresis Quality Score at the University of Miami.

Samples from the DarDar vaccine trial (n = 304) and the Household Contact Study (n = 263) were submitted for genotyping at the Hussman Institute for Human Genomics, Miami, Florida. A total of 567 samples passed quality control measures and were genotyped using the Illumina Human Core Exome Beadchip (542,585 SNPs). SNPs with a genotyping call rate < 0.95 and a Hardy-Weinberg equilibrium p-value < 1x10^-4^ were excluded. Participants with a per individual genotyping call rate < 0.95 were excluded. Concordance of reported and genotypic sex was verified. In case of relatedness among study participants (pi-hat > 0.20), one individual was randomly removed. The final study population included 270 participants from the extended follow up of the DarDar vaccine trial and 199 participants from the Household Contact Study. All quality control analyses were performed in PLINK(v1.07)[53]. Results for the most significant SNP are shown in S8 Fig.

### Statistical analyses

#### Statistical genetics methods

To adjust for possible admixture within each cohort, principal components were calculated using SNPs with r^2^ < 0.1 and MAF > 0.2 using the SNPRelate package in R[54, 55]. The qqman package in R was used to generate Manhattan and qq plots[56]. Locus zoom was used to plot the regions with the strongest association[57].

TST data were evaluated using additive, dominant, and recessive genetic models both as a continuous variable using linear regression, and as a binary variable (TST positive, ≥ 5mm vs. TST negative, < 5mm) with logistic regression in PLINK(v1.07)[53]. A total of 162,228 SNPs passed the inclusion criteria at a MAF > 0.20 (chosen to provide adequate power in our studies using QUANTO[58], S19 Table), corresponding to a Bonferroni corrected multiple testing threshold of 3.08x10^-7^. Summary statistics and univariate logistic regression models of TST^+^/TST^-^ status with available covariates were calculated in STATA(v11.2)[59]. Covariates associating with TST positivity (0.05 level) were included in final models.

All analyses were adjusted for 10 principal components to account for possible population structure, and analyses of the cohorts combined were adjusted for a cohort variable. SNPs in the regions of interest were imputed with IMPUTE2 (v2.3.1), using one phased reference panel from the 1000 Genomes project[60–62]. We used UNPHASED(v3.1.7)[63] to perform 2 SNP haplotype association analyses, adjusting for the same covariates as in the single SNP association analyses above. We studied all pairwise haplotypes that included the most significantly associating SNP in the combined cohort and an additional 30 available SNPs within 250 kb of this SNP that had a minor allele frequency >0.05. Haplotype plots were generated using Haploview[64].

#### Additional analyses to account for potential anergy

To help mitigate the confounding influence of anergy, we utilized available IFN-γ data. Subjects can remain TST-negative given an exposure to *M*. *tuberculosis* in multiple ways that may need to be accounted for analytically. MTB infection can be of an insufficient dose, inhaled but mechanically prevented from seeding the lungs, seed the lungs but be cleared before immune memory is invoked, potentially localized without a systemic response, and importantly for our analyses MTB can establish a latent infection but host immunosuppression and an inability to mount a delayed type hypersensitivity response can prevent a positive TST test, i.e. anergy[65]. To adjust for possible anergy in Tanzania, we removed all patients who had negative IFN-γ responses to the positive control antigen, PHA (defined as a PHA < 300pg/mL), which indicates low T cell counts or activity and lymphocyte proliferation assays (LPA) (defined as a proliferative index < 3), indicating few T cells exist in the individual. LPA data was not available for the Ugandan cohort; therefore, we removed all patients who had negative responses to all available antigens and to PHA (PHA < 300pg/mL). Patients who were PHA positive but negative for all other antigens remained in the study. ELISA assays were not performed on 102 patients from the HHC cohort and 33 patients from the DarDar vaccine trial extended follow up. Of the patients with missing assays, 71 had TST measurements ≥ 5mm, and 31 < 5mm in Uganda, and 10 had TST ≥ 5mm, and 23 < 5mm in Tanzania. Logistic regression models of TST status adjusting for missing ELISA data were performed to prevent confounding by missing data. Patients who stated that they had previous active TB, but had a TST of 0mm were excluded from the analyses presented below.

To evaluate possible false negative TST responses on our association results, additional logistic regression analyses were performed removing individuals with a 0mm TST induration who had a substantial IFN-γ response (> mean in TST positives) to any of the tested antigens at the time of the TST induration measurement (S20 Table). The effect of possible false positive TST results due to BCG vaccination was evaluated by performing logistic regression analyses removing individuals with positive TST scores but low ELISA response (< mean of TST negatives) to any of the tested antigens. Numbers of individuals excluded from the analyses and the criteria are presented in S21 Table.

#### Functional annotation

The ENCODE Project[66] was accessed via the UCSC Genome Browser[22] and used for functional annotation.

### Ethics

Informed consent was obtained from all patients in the extended DarDar follow-up cohort. The research ethics committee at the Muhimbili University of Health and Allied Sciences and the Committee for the Protection of Human Subjects at Dartmouth College and the Dartmouth-Hitchcock Medical Center approved this study. Informed consent was obtained from all subjects in the Household Contact study in Kampala, Uganda. Ethics committees that approved this work were at Muhimbili University of Health and Allied Sciences, Committee for the Protection of Human Subjects at Dartmouth College (#14606), Uganda Council for Science and Technology, and University Hospitals of Cleveland (10-01-25)

## Supporting information

S1 Table Single nucleotide polymorphisms associating with continuous tuberculin skin test in a dominant genetic model. (DOCX)

S2 Table Single nucleotide polymorphisms associating with dichotomous tuberculin skin test status in an additive genetic model. (DOCX)

S3 Table Single nucleotide polymorphisms associating with continuous tuberculin skin test induration in an additive genetic model. (DOCX)

S4 Table Single nucleotide polymorphisms associating with dichotomous tuberculin skin test status in a recessive genetic model. (DOCX)

S5 Table Single nucleotide polymorphisms associating with continuous tuberculin skin test in a recessive genetic model. (DOCX)

S6 Table Association of SNPs with dichotomous tuberculin skin test in the imputed SLC25A48/IL9 region. (DOCX)

S7 Table Association of SNPs with continuous tuberculin skin test induration in the imputed *SLC25A48/IL9* region. (DOCX)

S8 Table Most significant SNPs associating with dichotomous TST status in the subset of subjects for whom CD4 counts were available. (DOCX)

S9 Table Interferon gamma release assay results by TST case/control status. (DOCX)

S10 Table Tuberculin skin test reactivity data at enrollment from the entire Ugandan household contact study. (DOCX)

S11 Table Single nucleotide polymorphisms associating with tuberculin skin test dichotomous status (< versus ≥ 5mm) and continuous tuberculin skin test induration using a dominant genetic model in the combined cohort after removing patients with possible false negative TST results. (DOCX)

S12 Table Single nucleotide polymorphisms associating with tuberculin skin test dichotomous status (< versus ≥ 5mm) and continuous tuberculin skin test induration using a dominant genetic model in the combined cohort after removing patients with possible false positive TST reaction to a childhood BCG vaccine. (DOCX)

S13 Table Single nucleotide polymorphisms associating with tuberculin skin test dichotomous status (< versus ≥ 5mm) and continuous tuberculin skin test induration using a dominant genetic model in the combined cohort after removing patients with possible false positive TST reaction to a childhood BCG vaccine and possible false negative TST reactions. (DOCX)

S14 Table Association of SNPs with dichotomous tuberculin skin test status (< versus ≥ 5mm) and continuous tuberculin skin test induration in the combined cohort using a dominant genetic model. (DOCX)

S15 Table Association of SNPs with dichotomous tuberculin skin test status (< versus ≥ 5mm) and continuous tuberculin skin test induration using a dominant genetic model in the combined cohort, including patients with prior TB. (DOCX)

S16 Table Association of the rs877356-rs2069885 haplotype using additive genetic models for both SNPs with TST dichotomous status in the *SLC25A48/IL9* region. (DOCX)

S17 Table Analyses of available and imputed variants using continuous TST scores in the *SLC6A3* region, chromosome 5p15.33 and binary TST scores, most significant SNPs chromosome 11p14, chromosome 2q14, chromosome 2q21-q24, chromosome 5p13-q22, and chromosome 1q32.1 near *IL-10*. (DOCX)

S18 Table Single nucleotide polymorphisms associating with TST status near *GAS2* as previously linked to 11p14-15. (DOCX)

S19 Table Power calculation using a log additive model. (DOCX)

S20 Table Sample sizes for analyses accounting for anergy. (DOCX)

S21 Table Exclusions following quality control. (DOCX)

S1 Fig QQ plot of results from a logistic regression association of tuberculin skin test status. (DOCX)

S2 Fig Manhattan plot of results from a logistic regression association of case/control tuberculin skin test induration status (< versus ≥ 5mm) with a dominant genetic model. (DOCX)

S3 Fig Locus zoom plot of results from a logistic regression of case/control tuberculin skin test induration status (< versus ≥ 5mm) with SNPs in the *SLC25A48/IL9* region using a dominant genetic model in the combined cohort. (DOCX)

S4 Fig Locus zoom plot of results from a logistic regression association of dichotomous tuberculin skin test status (< versus ≥ 5mm) with a dominant genetic model of SNPs in the *SLC25A48/IL9* region in the Ugandan cohort. (DOCX)

S5 Fig Locus zoom plot of results from a logistic regression association of case/control tuberculin skin test induration status (< versus ≥ 5mm) with a dominant genetic model of SNPs in the *SLC25A48/IL9* region in the Tanzanian cohort. (DOCX)

S6 Fig Haploview plots of the rs877356 region using the D’ metric. (DOCX)

S7 Fig Haploview plots of the rs877356 region using the r2 metric. (DOCX)

S8 Fig Cluster plot for rs877356. Cases and controls were run together to minimize batch effects.
(DOCX)

## References

[1] Programme WHOGT. Global tuberculosis report 2014. 2014.

[2] Raviglione MC, Snider DE Jr., Kochi A. Global epidemiology of tuberculosis. Morbidity and mortality of a worldwide epidemic. Jama. 1995;273(3):220–6. . 7807661

[3] Stewart GR, Robertson BD, Young DB. Tuberculosis: a problem with persistence. Nature reviews Microbiology. 2003;1(2):97–105. doi: 10.1038/nrmicro749 . 15035039 10.1038/nrmicro749

[4] UNAIDS. UNAIDS report on the global AIDS epidemic. 2013.

[5] Selwyn PA, Schoenbaum EE, Davenny K, Robertson VJ, Feingold AR, Shulman JF, et al Prospective study of human immunodeficiency virus infection and pregnancy outcomes in intravenous drug users. Jama. 1989;261(9):1289–94. Epub 1989/03/03. . 2915455

[6] Di Perri G, Cruciani M, Danzi MC, Luzzati R, De Checchi G, Malena M, et al Nosocomial epidemic of active tuberculosis among HIV-infected patients. Lancet. 1989;2(8678–8679):1502–4. Epub 1989/12/23. . 2574778

[7] Rieder HL. Epidemiologic Basis of Tuberculosis Control. International Union Against Tuberculosis and Lung Disease, Paris. 1999:162.

[8] Cobat A, Gallant CJ, Simkin L, Black GF, Stanley K, Hughes J, et al Two loci control tuberculin skin test reactivity in an area hyperendemic for tuberculosis. The Journal of experimental medicine. 2009;206(12):2583–91. doi: 10.1084/jem.20090892 ; 19901083 10.1084/jem.20090892 PMC2806605

[9] Rose DN, Schechter CB, Adler JJ. Interpretation of the tuberculin skin test. Journal of general internal medicine. 1995;10(11):635–42. . 8583267 10.1007/BF02602749

[10] Ma N, Zalwango S, Malone LL, Nsereko M, Wampande EM, Thiel BA, et al Clinical and epidemiological characteristics of individuals resistant to M. tuberculosis infection in a longitudinal TB household contact study in Kampala, Uganda. BMC infectious diseases. 2014;14:352 doi: 10.1186/1471-2334-14-352 ; 24970328 10.1186/1471-2334-14-352 PMC4091673

[11] Kumar V AA, Fausto N, Aster JC. Robbins & Cotran Pathologic Basis of Disease. 8th ed: Saunders 2010.

[12] Vukmanovic-Stejic M, Reed JR, Lacy KE, Rustin MH, Akbar AN. Mantoux Test as a model for a secondary immune response in humans. Immunology letters. 2006;107(2):93–101. doi: 10.1016/j.imlet.2006.08.002 . 16979761 10.1016/j.imlet.2006.08.002

[13] Sepulveda RL, Heiba IM, King A, Gonzalez B, Elston RC, Sorensen RU. Evaluation of tuberculin reactivity in BCG-immunized siblings. American journal of respiratory and critical care medicine. 1994;149(3 Pt 1):620–4. doi: 10.1164/ajrccm.149.3.8118628 . 8118628 10.1164/ajrccm.149.3.8118628

[14] Sepulveda RL, Heiba IM, Navarrete C, Elston RC, Gonzalez B, Sorensen RU. Tuberculin reactivity after newborn BCG immunization in mono- and dizygotic twins. Tuber Lung Dis. 1994;75(2):138–43. doi: 10.1016/0962-8479(94)90043-4 . 8032047 10.1016/0962-8479(94)90043-4

[15] Stein CM, Zalwango S, Malone LL, Won S, Mayanja-Kizza H, Mugerwa RD, et al Genome scan of M. tuberculosis infection and disease in Ugandans. PloS one. 2008;3(12):e4094 doi: 10.1371/journal.pone.0004094 ; 19116662 10.1371/journal.pone.0004094 PMC2605555

[16] CDC. Interferon-Gamma Release Assays (IGRAs)—Blood Tests for TB Infection 2011. Available from: http://www.cdc.gov/tb/publications/factsheets/testing/IGRA.html.

[17] Gold CQ-T. [Feb 2015]. Available from: http://www.cellestis.com/IRM/content/aust/qtfproducts_tbgoldintube.html.

[18] Farhat M, Greenaway C, Pai M, Menzies D. False-positive tuberculin skin tests: what is the absolute effect of BCG and non-tuberculous mycobacteria? Int J Tuberc Lung Dis. 2006;10(11):1192–204. . 17131776

[19] Sobota RS, Stein CM, Kodaman N, Scheinfeldt LB, Maro I, Wieland-Alter W, et al A Locus at 5q33.3 Confers Resistance to Tuberculosis in Highly Susceptible Individuals. Am J Hum Genet. 2016;98(3):514–24. doi: 10.1016/j.ajhg.2016.01.015 ; 26942285 10.1016/j.ajhg.2016.01.015 PMC4800052

[20] Hall NB, Igo RP Jr., Malone LL, Truitt B, Schnell A, Tao L, et al Polymorphisms in TICAM2 and IL1B are associated with TB. Genes Immun. 2015;16(2):127–33. doi: 10.1038/gene.2014.77 ; 25521228 10.1038/gene.2014.77 PMC4352113

[21] Cobat A, Poirier C, Hoal E, Boland-Auge A, de La Rocque F, Corrard F, et al Tuberculin skin test negativity is under tight genetic control of chromosomal region 11p14-15 in settings with different tuberculosis endemicities. J Infect Dis. 2015;211(2):317–21. doi: 10.1093/infdis/jiu446 ; 25143445 10.1093/infdis/jiu446 PMC4279780

[22] Fujita PA, Rhead B, Zweig AS, Hinrichs AS, Karolchik D, Cline MS, et al The UCSC Genome Browser database: update 2011. Nucleic acids research. 2011;39(Database issue):D876–82. doi: 10.1093/nar/gkq963 ; 20959295 10.1093/nar/gkq963 PMC3242726

[23] Liu X, Cheng R, Verbitsky M, Kisselev S, Browne A, Mejia-Sanatana H, et al Genome-wide association study identifies candidate genes for Parkinson's disease in an Ashkenazi Jewish population. BMC medical genetics. 2011;12:104 doi: 10.1186/1471-2350-12-104 ; 21812969 10.1186/1471-2350-12-104 PMC3166909

[24] Palmieri F. The mitochondrial transporter family SLC25: identification, properties and physiopathology. Molecular aspects of medicine. 2013;34(2–3):465–84. doi: 10.1016/j.mam.2012.05.005 . 23266187 10.1016/j.mam.2012.05.005

[25] Postma DS, Bleecker ER, Amelung PJ, Holroyd KJ, Xu J, Panhuysen CI, et al Genetic susceptibility to asthma—bronchial hyperresponsiveness coinherited with a major gene for atopy. The New England journal of medicine. 1995;333(14):894–900. doi: 10.1056/NEJM199510053331402 . 7666875 10.1056/NEJM199510053331402

[26] Hopp RJ, Townley RG, Biven RE, Bewtra AK, Nair NM. The presence of airway reactivity before the development of asthma. The American review of respiratory disease. 1990;141(1):2–8. doi: 10.1164/ajrccm/141.1.2 . 2404438 10.1164/ajrccm/141.1.2

[27] Longo G, Strinati R, Poli F, Fumi F. Genetic factors in nonspecific bronchial hyperreactivity. An epidemiologic study. American journal of diseases of children. 1987;141(3):331–4. . 3812414 10.1001/archpedi.1987.04460030109037

[28] Shimwela M, Mwita JC, Mwandri M, Rwegerera GM, Mashalla Y, Mugusi F. Asthma prevalence, knowledge, and perceptions among secondary school pupils in rural and urban coastal districts in Tanzania. BMC public health. 2014;14:387 doi: 10.1186/1471-2458-14-387 ; 24754895 10.1186/1471-2458-14-387 PMC4023699

[29] von Hertzen L, Klaukka T, Mattila H, Haahtela T. Mycobacterium tuberculosis infection and the subsequent development of asthma and allergic conditions. The Journal of allergy and clinical immunology. 1999;104(6):1211–4. . 10589003 10.1016/s0091-6749(99)70015-1

[30] von Mutius E, Pearce N, Beasley R, Cheng S, von Ehrenstein O, Bjorksten B, et al International patterns of tuberculosis and the prevalence of symptoms of asthma, rhinitis, and eczema. Thorax. 2000;55(6):449–53. ; doi: 10.1136/thorax.55.6.449 10817790 10.1136/thorax.55.6.449 PMC1745787

[31] Goswami R, Kaplan MH. A brief history of IL-9. Journal of immunology. 2011;186(6):3283–8. doi: 10.4049/jimmunol.1003049 ; 21368237 10.4049/jimmunol.1003049 PMC3074408

[32] Temann UA, Laouar Y, Eynon EE, Homer R, Flavell RA. IL9 leads to airway inflammation by inducing IL13 expression in airway epithelial cells. International immunology. 2007;19(1):1–10. doi: 10.1093/intimm/dxl117 . 17101709 10.1093/intimm/dxl117

[33] Uyttenhove C, Simpson RJ, Van Snick J. Functional and structural characterization of P40, a mouse glycoprotein with T-cell growth factor activity. Proceedings of the National Academy of Sciences of the United States of America. 1988;85(18):6934–8. ; 3137580 10.1073/pnas.85.18.6934 PMC282093

[34] Dugas B, Renauld JC, Pene J, Bonnefoy JY, Peti-Frere C, Braquet P, et al Interleukin-9 potentiates the interleukin-4-induced immunoglobulin (IgG, IgM and IgE) production by normal human B lymphocytes. European journal of immunology. 1993;23(7):1687–92. doi: 10.1002/eji.1830230743 . 7686859 10.1002/eji.1830230743

[35] Petit-Frere C, Dugas B, Braquet P, Mencia-Huerta JM. Interleukin-9 potentiates the interleukin-4-induced IgE and IgG1 release from murine B lymphocytes. Immunology. 1993;79(1):146–51. ; 8509135 PMC1422055

[36] Sears MR, Burrows B, Flannery EM, Herbison GP, Hewitt CJ, Holdaway MD. Relation between airway responsiveness and serum IgE in children with asthma and in apparently normal children. The New England journal of medicine. 1991;325(15):1067–71. doi: 10.1056/NEJM199110103251504 . 1891008 10.1056/NEJM199110103251504

[37] Donahue RE, Yang YC, Clark SC. Human P40 T-cell growth factor (interleukin-9) supports erythroid colony formation. Blood. 1990;75(12):2271–5. . 1693525

[38] Williams DE, Morrissey PJ, Mochizuki DY, de Vries P, Anderson D, Cosman D, et al T-cell growth factor P40 promotes the proliferation of myeloid cell lines and enhances erythroid burst formation by normal murine bone marrow cells in vitro. Blood. 1990;76(5):906–11. . 2118397

[39] Longphre M, Li D, Gallup M, Drori E, Ordonez CL, Redman T, et al Allergen-induced IL-9 directly stimulates mucin transcription in respiratory epithelial cells. The Journal of clinical investigation. 1999;104(10):1375–82. doi: 10.1172/JCI6097 ; 10562299 10.1172/JCI6097 PMC409835

[40] Yamasaki A, Saleh A, Koussih L, Muro S, Halayko AJ, Gounni AS. IL-9 induces CCL11 expression via STAT3 signalling in human airway smooth muscle cells. PloS one. 2010;5(2):e9178 doi: 10.1371/journal.pone.0009178 ; 20169197 10.1371/journal.pone.0009178 PMC2820544

[41] Cambier CJ, Falkow S, Ramakrishnan L. Host evasion and exploitation schemes of Mycobacterium tuberculosis. Cell. 2014;159(7):1497–509. doi: 10.1016/j.cell.2014.11.024 . 25525872 10.1016/j.cell.2014.11.024

[42] Seshadri C, Uiso LO, Ostermann J, Diefenthal H, Shao HJ, Chu HY, et al Low sensitivity of T-cell based detection of tuberculosis among HIV co-infected Tanzanian in-patients. East Afr Med J. 2008;85(9):442–9. ; 19537417 10.4314/eamj.v85i9.117085 PMC3168735

[43] Whalen CC, Zalwango S, Chiunda A, Malone L, Eisenach K, Joloba M, et al Secondary attack rate of tuberculosis in urban households in Kampala, Uganda. PloS one. 2011;6(2):e16137 Epub 2011/02/23. doi: 10.1371/journal.pone.0016137 ; 21339819 10.1371/journal.pone.0016137 PMC3038854

[44] Siezen CL, Bont L, Hodemaekers HM, Ermers MJ, Doornbos G, Van't Slot R, et al Genetic susceptibility to respiratory syncytial virus bronchiolitis in preterm children is associated with airway remodeling genes and innate immune genes. The Pediatric infectious disease journal. 2009;28(4):333–5. Epub 2009/03/05. doi: 10.1097/INF.0b013e31818e2aa9 . 19258923 10.1097/INF.0b013e31818e2aa9

[45] Janssen R, Bont L, Siezen CL, Hodemaekers HM, Ermers MJ, Doornbos G, et al Genetic susceptibility to respiratory syncytial virus bronchiolitis is predominantly associated with innate immune genes. J Infect Dis. 2007;196(6):826–34. Epub 2007/08/19. doi: 10.1086/520886 . 17703412 10.1086/520886

[46] von Reyn CF, Mtei L, Arbeit RD, Waddell R, Cole B, Mackenzie T, et al Prevention of tuberculosis in Bacille Calmette-Guerin-primed, HIV-infected adults boosted with an inactivated whole-cell mycobacterial vaccine. Aids. 2010;24(5):675–85. Epub 2010/02/02. doi: 10.1097/QAD.0b013e3283350f1b . 20118767 10.1097/QAD.0b013e3283350f1b PMC10525041

[47] Stein CM, Hall NB, Malone LL, Mupere E. The household contact study design for genetic epidemiological studies of infectious diseases. Front Genet. 2013;4:61 Epub 2013/05/04. doi: 10.3389/fgene.2013.00061 ; 23641253 10.3389/fgene.2013.00061 PMC3639375

[48] Hall NB, Igo RP Jr., Malone LL, Truitt B, Schnell A, Tao L, et al Polymorphisms in TICAM2 and IL1B are associated with TB. Genes Immun. 2014 Epub 2014/12/19. doi: 10.1038/gene.2014.77 . 25521228 10.1038/gene.2014.77 PMC4352113

[49] Blumberg HM, Burman WJ, Chaisson RE, Daley CL, Etkind SC, Friedman LN, et al American Thoracic Society/Centers for Disease Control and Prevention/Infectious Diseases Society of America: treatment of tuberculosis. American journal of respiratory and critical care medicine. 2003;167(4):603–62. Epub 2003/02/18. doi: 10.1164/rccm.167.4.603 . 12588714 10.1164/rccm.167.4.603

[50] Targeted tuberculin testing and treatment of latent tuberculosis infection. American Thoracic Society. MMWR Recomm Rep. 2000;49(RR-6):1–51. . 10881762

[51] Lahey T, Mitchell BK, Arbeit RD, Sheth S, Matee M, Horsburgh CR, et al Polyantigenic interferon-gamma responses are associated with protection from TB among HIV-infected adults with childhood BCG immunization. PloS one. 2011;6(7):e22074 doi: 10.1371/journal.pone.0022074 ; 21799772 10.1371/journal.pone.0022074 PMC3140474

[52] Mahan CS, Zalwango S, Thiel BA, Malone LL, Chervenak KA, Baseke J, et al Innate and adaptive immune responses during acute M. tuberculosis infection in adult household contacts in Kampala, Uganda. The American journal of tropical medicine and hygiene. 2012;86(4):690–7. doi: 10.4269/ajtmh.2012.11-0553 ; 22492155 10.4269/ajtmh.2012.11-0553 PMC3403758

[53] Purcell S, Neale B, Todd-Brown K, Thomas L, Ferreira MA, Bender D, et al PLINK: a tool set for whole-genome association and population-based linkage analyses. Am J Hum Genet. 2007;81(3):559–75. Epub 2007/08/19. doi: 10.1086/519795 ; 17701901 10.1086/519795 PMC1950838

[54] RDC T. R: A language and environment for statistical computing. Vienna: R Foundation for Statistical Computing. 2007.

[55] Zheng X, Levine D, Shen J, Gogarten SM, Laurie C, Weir BS. A high-performance computing toolset for relatedness and principal component analysis of SNP data. Bioinformatics. 2012;28(24):3326–8. Epub 2012/10/13. doi: 10.1093/bioinformatics/bts606 ; 23060615 10.1093/bioinformatics/bts606 PMC3519454

[56] Turner SD. qqman: an R package for visualizing GWAS results using Q-Q and manhattan plots. bioRxiv beta. 2014 doi: 10.1101/005165

[57] Pruim RJ, Welch RP, Sanna S, Teslovich TM, Chines PS, Gliedt TP, et al LocusZoom: regional visualization of genome-wide association scan results. Bioinformatics. 2010;26(18):2336–7. doi: 10.1093/bioinformatics/btq419 ; 20634204 10.1093/bioinformatics/btq419 PMC2935401

[58] Gauderman WJ. Sample size requirements for matched case-control studies of gene-environment interaction. Stat Med. 2002;21(1):35–50. Epub 2002/01/10. . 11782049 10.1002/sim.973

[59] StataCorp. Stata Statistical Software: Release 11. College Station, TX: StataCorp LP; 2009.

[60] Genomes Project C, Abecasis GR, Auton A, Brooks LD, DePristo MA, Durbin RM, et al An integrated map of genetic variation from 1,092 human genomes. Nature. 2012;491(7422):56–65. Epub 2012/11/07. doi: 10.1038/nature11632 ; 23128226 10.1038/nature11632 PMC3498066

[61] Howie BN, Donnelly P, Marchini J. A flexible and accurate genotype imputation method for the next generation of genome-wide association studies. PLoS genetics. 2009;5(6):e1000529 Epub 2009/06/23. doi: 10.1371/journal.pgen.1000529 ; 19543373 10.1371/journal.pgen.1000529 PMC2689936

[62] Howie B, Marchini J, Stephens M. Genotype imputation with thousands of genomes. G3. 2011;1(6):457–70. Epub 2012/03/03. doi: 10.1534/g3.111.001198 ; 22384356 10.1534/g3.111.001198 PMC3276165

[63] Dudbridge F. Likelihood-based association analysis for nuclear families and unrelated subjects with missing genotype data. Human heredity. 2008;66(2):87–98. Epub 2008/04/03. doi: 10.1159/000119108 ; 18382088 10.1159/000119108 PMC2386559

[64] Barrett JC, Fry B, Maller J, Daly MJ. Haploview: analysis and visualization of LD and haplotype maps. Bioinformatics. 2005;21(2):263–5. Epub 2004/08/07. doi: 10.1093/bioinformatics/bth457 . 15297300 10.1093/bioinformatics/bth457

[65] O'Garra A, Redford PS, McNab FW, Bloom CI, Wilkinson RJ, Berry MP. The immune response in tuberculosis. Annu Rev Immunol. 2013;31:475–527. doi: 10.1146/annurev-immunol-032712-095939 . 23516984 10.1146/annurev-immunol-032712-095939

[66] Consortium EP. An integrated encyclopedia of DNA elements in the human genome. Nature. 2012;489(7414):57–74. doi: 10.1038/nature11247 . 22955616 10.1038/nature11247 PMC3439153

