# Supplementary material for: A chromosome 5q31.1 locus associates with tuberculin skin test reactivity in HIV-positive individuals from tuberculosis hyper-endemic regions in east Africa
Source: PLoS Genet. 2017 Jun 19;13(6):e1006710. doi: 10.1371/journal.pgen.1006710 (PMC5495514; doi:10.1371/journal.pgen.1006710)
Supplement: S1 Table — (DOCX) [file pgen.1006710.s001.docx]

**S1 Table.** Single nucleotide polymorphisms associating with continuous tuberculin skin test below a 5x10^-5^ p value in a dominant genetic model in the combined cohort*, the Ugandan cohort^, and the Tanzanian cohort^

| Combined Cohort | | | | | | | | |
| --- | --- | --- | --- | --- | --- | --- | --- | --- |
| SNP | Chr. | Minor Allele | MAF | n | Beta | 95% Confidence Interval | p value | Nearest gene |
| rs877356 | 5 | T | 0.2292 | 469 | -4.144 | (-5.552, -2.737) | 1.45E-08 | *SLC25A48/IL9* |
| rs7239554 | 18 | A | 0.2804 | 469 | -3.013 | (-4.437, -1.59) | 3.97E-05 | *C18orf10* |
| rs6974557 | 7 | T | 0.2623 | 469 | -2.984 | (-4.395, -1.573) | 4.05E-05 | *Loc646614* |
| rs6733728 | 2 | C | 0.3838 | 469 | -3.044 | (-4.486, -1.603) | 4.13E-05 | *Loc402093* |
| rs2389096 | 13 | T | 0.2382 | 468 | 3.028 | (1.584, 4.472) | 4.69E-05 | *GPC6* |
| rs267280 | 3 | G | 0.468 | 469 | -3.290 | (-4.859, -1.721) | 4.70E-05 | *LARS2* |
| Ugandan Cohort | | | | | | | | |
| rs7326145 | 13 | A | 0.2337 | 199 | 4.953 | (2.815, 7.091) | 1.01E-05 | *COL4A2* |
| rs7837658 | 8 | T | 0.4472 | 199 | 5.124 | (2.856, 7.392) | 1.62E-05 | *RNF19A* |
| rs877356 | 5 | T | 0.2337 | 199 | -4.717 | (-6.819, -2.615) | 1.84E-05 | *SLC25A48/IL9* |
| rs7944514 | 11 | C | 0.4121 | 199 | 4.825 | (2.654, 6.996) | 2.19E-05 | *POLD3* |
| rs2839520 | 21 | A | 0.2663 | 199 | 4.452 | (2.378, 6.526) | 4.01E-05 | *UBASH3A* |
| rs16872344 | 5 | A | 0.2211 | 199 | 4.512 | (2.396, 6.629) | 4.51E-05 | *IRX1* |
| rs13174381 | 5 | A | 0.2312 | 199 | 4.474 | (2.366, 6.582) | 4.86E-05 | *IRX1* |
| rs10085086 | 5 | C | 0.2005 | 197 | -4.762 | (-7.006, -2.518) | 4.90E-05 | *Loc391738* |
| Tanzanian Cohort | | | | | | | | |
| rs17062122 | 6 | C | 0.3259 | 270 | -4.691 | (-6.56, -2.823) | 1.54E-06 | *Loc285735* |
| rs903281 | 10 | G | 0.2926 | 270 | -4.358 | (-6.21, -2.506) | 6.28E-06 | *RAB18* |
| rs331086 | 5 | C | 0.4648 | 270 | -4.577 | (-6.668, -2.487) | 2.52E-05 | *FBN2* |
| rs7137335 | 12 | T | 0.3327 | 269 | -4.022 | (-5.894, -2.15) | 3.53E-05 | *SLC16A7* |
| rs7074813 | 10 | G | 0.3278 | 270 | -4.016 | (-5.895, -2.138) | 3.84E-05 | *RAB18* |
| … | … | … | … | … | … | … | … | *…* |
| rs877356 | 5 | T | 0.2259 | 270 | -3.592 | (-5.516, -1.667) | 3.09E-04 | *SLC25A48/IL9* |

* adjusted for 10 principal components, sex, and cohort of origin

^ adjusted for 10 principal components and sex
