# Supplementary material for: A chromosome 5q31.1 locus associates with tuberculin skin test reactivity in HIV-positive individuals from tuberculosis hyper-endemic regions in east Africa
Source: PLoS Genet. 2017 Jun 19;13(6):e1006710. doi: 10.1371/journal.pgen.1006710 (PMC5495514; doi:10.1371/journal.pgen.1006710)
Supplement: S2 Table — (DOCX) [file pgen.1006710.s002.docx]

**S2 Table.** Single nucleotide polymorphisms associating with dichotomous tuberculin skin test status (< versus ≥ 5mm) below a 5x10^-5^ p value in an additive genetic model in the combined cohort*, the Ugandan cohort^, and the Tanzanian cohort^

| Combined Cohort | | | | | | | | | |
| --- | --- | --- | --- | --- | --- | --- | --- | --- | --- |
| SNP | Chr. | Minor Allele | MAF | n | Odds Ratio | 95% Confidence Interval | p value | | Nearest gene |
| rs877356 | 5 | T | 0.2292 | 469 | 0.331 | (0.222, 0.492) | 5.45E-08 | | *SLC25A48/IL9* |
| rs7808481 | 7 | A | 0.2164 | 469 | 2.311 | (1.598, 3.343) | 8.67E-06 | | *Loc340268* |
| rs10804666 | 3 | G | 0.4392 | 469 | 2.042 | (1.467, 2.841) | 2.28E-05 | | *NMNAT3* |
| rs8179938 | 3 | A | 0.3731 | 469 | 2.011 | (1.451, 2.788) | 2.77E-05 | | *Loc643634* |
| rs4705073 | 5 | C | 0.4499 | 469 | 1.914 | (1.413, 2.593) | 2.79E-05 | | *MIRN145* |
| rs964739 | 4 | T | 0.3795 | 469 | 1.928 | (1.412, 2.634) | 3.67E-05 | | *KLHL8* |
| Ugandan Cohort | | | | | | | | | |
| rs877356 | 5 | T | 0.2337 | 199 | 0.228 | (0.117, 0.444) | 1.41E-05 | | *SLC25A48/IL9* |
| rs9989936 | 21 | G | 0.402 | 199 | 0.251 | (0.131, 0.481) | | 3.07E-05 | *SAMSN1* |
| Tanzanian Cohort | | | | | | | | | |
| rs642774 | 1 | G | 0.4407 | 270 | 0.371 | (0.242, 0.571) | | 6.26E-06 | *UOX* |
| rs6589880 | 11 | C | 0.4037 | 270 | 2.232 | (1.520, 3.280) | | 4.18E-05 | *Loc283155* |
| rs7808481 | 7 | A | 0.2315 | 270 | 2.529 | (1.618, 3.955) | | 4.74E-05 | *Loc340268* |
| … | … | … | … | … | … | … | | … | *…* |
| rs877356 | 5 | T | 0.2259 | 270 | 0.383 | (0.228, 0.646) | | 3.14E-04 | *SLC25A48/IL9* |

* adjusted for 10 principal components, sex, and cohort of origin

^ adjusted for 10 principal components and sex
