# Supplementary material for: A chromosome 5q31.1 locus associates with tuberculin skin test reactivity in HIV-positive individuals from tuberculosis hyper-endemic regions in east Africa
Source: PLoS Genet. 2017 Jun 19;13(6):e1006710. doi: 10.1371/journal.pgen.1006710 (PMC5495514; doi:10.1371/journal.pgen.1006710)
Supplement: S3 Table — (DOCX) [file pgen.1006710.s003.docx]

**S3 Table.** Single nucleotide polymorphisms associating with continuous tuberculin skin test induration below a 5x10^-5^ p value in an additive genetic model in the combined cohort*, the Ugandan cohort^, and the Tanzanian cohort^

| Combined Cohort | | | | | | | | |
| --- | --- | --- | --- | --- | --- | --- | --- | --- |
| SNP | Chr. | Minor Allele | MAF | n | Beta | 95% Confidence Interval | p value | Nearest gene |
| rs877356 | 5 | T | 0.2292 | 469 | -3.336 | (-4.529, -2.144) | 6.95E-08 | *SLC25A48/IL9* |
| rs7082209 | 10 | G | 0.4019 | 469 | -2.563 | (-3.664, -1.463) | 6.45E-06 | *CXCL12* |
| rs7163504 | 15 | G | 0.4251 | 467 | -2.269 | (-3.262, -1.276) | 9.51E-06 | *NEO1* |
| rs8179938 | 3 | A | 0.3731 | 469 | 2.366 | (1.304, 3.428) | 1.56E-05 | *Loc643636* |
| rs10881240 | 1 | T | 0.3731 | 469 | -2.229 | (-3.249, -1.209) | 2.25E-05 | *Loc642337* |
| rs290185 | 11 | A | 0.4627 | 469 | 2.180 | (1.173, 3.186) | 2.66E-05 | *CCDC89* |
| rs7239554 | 18 | A | 0.2804 | 469 | -2.452 | (-3.588, -1.317) | 2.80E-05 | *C18orf10* |
| rs11120119 | 1 | G | 0.4506 | 466 | -2.148 | (-3.144, -1.152) | 2.89E-05 | *RPS6KC1* |
| rs7808481 | 7 | A | 0.2164 | 469 | 2.572 | (1.375, 3.769) | 3.05E-05 | *Loc340268* |
| rs6780136 | 3 | T | 0.3323 | 468 | -2.210 | (-3.239, -1.18) | 3.12E-05 | *CPNE4* |
| rs12634351 | 3 | C | 0.3294 | 469 | -2.214 | (-3.253, -1.176) | 3.50E-05 | *CPNE4* |
| rs10804666 | 3 | G | 0.4392 | 469 | 2.249 | (1.185, 3.312) | 4.06E-05 | *NMNAT3* |
| rs7326145 | 13 | A | 0.2495 | 469 | 2.484 | (1.305, 3.662) | 4.29E-05 | *COL4A2* |
| Ugandan Cohort | | | | | | | | |
| rs7326145 | 13 | A | 0.2337 | 199 | 4.296 | (2.442, 6.15) | 1.00E-05 | *COL4A2* |
| rs10085086 | 5 | C | 0.2005 | 197 | -4.213 | (-6.135, -2.292) | 2.80E-05 | *Loc391738* |
| rs6545560 | 2 | G | 0.402 | 199 | -3.204 | (-4.676, -1.732) | 3.15E-05 | *CCDC85A* |
| … | … | … | … | … | … | … | … | *…* |
| rs877356 | 5 | T | 0.2337 | 199 | 0.228 | (0.117, 0.444) | 1.25E-04 | *SLC25A48/IL9* |
| Tanzanian Cohort | | | | | | | | |
| rs642774 | 1 | G | 0.4407 | 270 | -3.102 | (-4.458, -1.745) | 1.11E-05 | *UOX* |
| rs2727529 | 7 | C | 0.3833 | 270 | 2.925 | (1.603, 4.247) | 2.07E-05 | *PRKAG2* |
| rs312305 | 5 | G | 0.4349 | 269 | 2.898 | (1.574, 4.223) | 2.55E-05 | *GABRG2* |
| rs2141372 | 2 | G | 0.3963 | 270 | -3.101 | (-4.52, -1.682) | 2.62E-05 | *ZNF512* |
| rs7808481 | 7 | A | 0.2315 | 270 | 3.350 | (1.803, 4.897) | 3.06E-05 | *Loc340268* |
| rs8103597 | 19 | A | 0.2611 | 270 | 3.255 | (1.741, 4.77) | 3.48E-05 | *SIPA1L3* |
| rs17062122 | 6 | C | 0.3259 | 270 | -3.131 | (-4.596, -1.666) | 3.85E-05 | *Loc285735* |
| rs12469734 | 2 | A | 0.4463 | 270 | 2.993 | (1.578, 4.407) | 4.58E-05 | *ARL4C* |
| rs7906180 | 10 | T | 0.3630 | 270 | 2.979 | (1.567, 4.391) | 4.81E-05 | *Loc100128641* |
| … | … | … | … | … | … | … | … | *…* |
| rs877356 | 5 | T | 0.2259 | 270 | -3.05 | (-4.646, -1.454) | 2.22E-04 | *SLC25A48/IL9* |

* adjusted for 10 principal components, sex, and cohort of origin

^ adjusted for 10 principal components and sex
