# Supplementary material for: A chromosome 5q31.1 locus associates with tuberculin skin test reactivity in HIV-positive individuals from tuberculosis hyper-endemic regions in east Africa
Source: PLoS Genet. 2017 Jun 19;13(6):e1006710. doi: 10.1371/journal.pgen.1006710 (PMC5495514; doi:10.1371/journal.pgen.1006710)
Supplement: S4 Table — (DOCX) [file pgen.1006710.s004.docx]

**S4 Table.** Single nucleotide polymorphisms associating with dichotomous tuberculin skin test status (< versus ≥ 5mm) below a 5x10^-5^ p value in a recessive genetic model in the combined cohort*, the Ugandan cohort^, and the Tanzanian cohort^

| Combined Cohort | | | | | | | | | |  |  |
| --- | --- | --- | --- | --- | --- | --- | --- | --- | --- | --- | --- |
| SNP | Chr. | Minor Allele | MAF | n | Odds Ratio | 95% Confidence Interval | | p value | Nearest gene |  |  |
| rs1293940 | 6 | A | 0.3166 | 469 | 0.172 | (0.0797, 0.371) | | 7.21E-06 | *ESR1* |  |  |
| rs2285513 | 19 | A | 0.4019 | 469 | 0.265 | (0.143, 0.492) | | 2.58E-05 | *SBSN* |  |  |
| rs2434785 | 5 | G | 0.4584 | 469 | 0.318 | (0.185, 0.548) | | 3.73E-05 | *Loc266786* |  |  |
| rs10804666 | 3 | G | 0.4392 | 469 | 3.569 | (1.946, 6.545) | | 3.94E-05 | *NMNAT3* |  |  |
| rs4705073 | 5 | C | 0.4499 | 469 | 2.990 | (1.771, 5.048) | | 4.14E-05 | *MIRN145* |  |  |
| rs2489772 | 1 | C | 0.4328 | 469 | 3.428 | (1.896, 6.196) | | 4.53E-05 | *KAZN* |  |  |
| Ugandan Cohort | | | | | | | | | |  |  |
| rs4989483 | 16 | G | 0.4925 | 199 | 0.1624 | (0.069, 0.381) | | 2.94E-05 | *FLJ32252* |  |  |
| rs12412686 | 10 | A | 0.3291 | 199 | 0.0905 | (0.028, 0.288) | 4.81E-05 | | *CLRN3* | |  |
| Tanzanian Cohort | | | | | | | | | | |  |
| rs7239336 | 18 | C | 0.4833 | 270 | 3.941 | (1.915, 6.366) | 4.52E-05 | |  | | *MIRN924* |

* adjusted for 10 principal components, sex, and cohort of origin

^ adjusted for 10 principal components and sex
