# Supplementary material for: A chromosome 5q31.1 locus associates with tuberculin skin test reactivity in HIV-positive individuals from tuberculosis hyper-endemic regions in east Africa
Source: PLoS Genet. 2017 Jun 19;13(6):e1006710. doi: 10.1371/journal.pgen.1006710 (PMC5495514; doi:10.1371/journal.pgen.1006710)
Supplement: S5 Table — (DOCX) [file pgen.1006710.s005.docx]

**S5 Table.** Single nucleotide polymorphisms associating with continuous tuberculin skin test below a 5x10^-5^ p value in a recessive genetic model in the combined cohort*, the Ugandan cohort^, and the Tanzanian cohort^

| Combined Cohort | | | | | | | | |
| --- | --- | --- | --- | --- | --- | --- | --- | --- |
| SNP | Chr. | Minor Allele | MAF | n | Beta | 95% Confidence Interval | p value | Nearest gene |
| rs2520696 | 13 | A | 0.468 | 469 | -4.000 | (-5.644, -2.356) | 2.51E-06 | *FRY* |
| rs2333021 | 14 | A | 0.3252 | 469 | 5.159 | (2.955, 7.362) | 5.77E-06 | *ZFYVE1* |
| rs10804666 | 3 | G | 0.4392 | 469 | 4.301 | (2.408, 6.194) | 1.07E-05 | *NMNAT3* |
| rs798957 | 13 | A | 0.3699 | 469 | -4.493 | (-6.478, -2.508) | 1.15E-05 | *FRY* |
| rs2434785 | 5 | G | 0.4584 | 469 | -3.882 | (-5.618, -2.146) | 1.46E-05 | *Loc266786* |
| rs2489772 | 1 | C | 0.4328 | 469 | 4.218 | (2.329, 6.107) | 1.50E-05 | *KAZN* |
| rs10957982 | 8 | T | 0.2564 | 468 | -6.268 | (-9.185, -3.352) | 3.05E-05 | *ZBTB10* |
| rs753927 | 20 | C | 0.3497 | 469 | 4.641 | (2.481, 6.801) | 3.07E-05 | *FERMT1* |
| rs2285513 | 19 | A | 0.4019 | 469 | -4.218 | (-6.191, -2.246) | 3.33E-05 | *SBSN* |
| rs1293940 | 6 | A | 0.3166 | 469 | -4.739 | (-6.962, -2.517) | 3.50E-05 | *ESR1* |
| rs10776801 | 1 | C | 0.3092 | 469 | -5.089 | (-7.476, -2.702) | 3.53E-05 | *Loc100130948* |
| rs10881240 | 1 | T | 0.3731 | 469 | -4.266 | (-6.274, -2.258) | 3.73E-05 | *Loc642337* |
| rs7623698 | 3 | A | 0.2388 | 469 | -6.577 | (-9.696, -3.458) | 4.27E-05 | *Loc285303* |
| Ugandan Cohort | | | | | | | | |
| rs10800363 | 1 | T | 0.4422 | 199 | -6.032 | (-8.773, -3.291) | 2.61E-05 | *XCL1* |
| rs8014986 | 14 | A | 0.2286 | 199 | -8.272 | (-12.04, -4.503) | 2.73E-05 | *HHIPL1* |
| rs7873440 | 9 | T | 0.4146 | 199 | -6.693 | (-9.8, -3.585) | 3.80E-05 | *RG9MTD3* |
| rs4989483 | 16 | G | 0.4925 | 199 | -5.509 | (-8.068, -2.951) | 3.81E-05 | *FLJ32252* |
| rs7676378 | 4 | T | 0.2538 | 199 | -8.992 | (-13.210, -4.769) | 4.60E-05 | *FAT4* |
| rs7340961 | 4 | C | 0.4095 | 199 | -6.079 | (-8.946, -3.212) | 4.94E-05 | *AGPAT9* |
| Tanzanian Cohort | | | | | | | | |
| rs2333021 | 14 | A | 0.3667 | 270 | 6.742 | (4.052, 9.433) | 1.61E-06 | *ZFYVE1* |
| rs4462385 | 12 | A | 0.2463 | 270 | 8.007 | (4.408, 11.61) | 1.88E-05 | *SLC16A7* |
| rs8019592 | 14 | T | 0.2037 | 270 | 11.530 | (6.302, 16.75) | 2.19E-05 | *RAD51L1* |
| rs2395015 | 7 | A | 0.2907 | 270 | 8.332 | (4.523, 12.14) | 2.56E-05 | *SMURF1* |
| rs5767477 | 22 | G | 0.3722 | 270 | 5.548 | (2.961, 8.135) | 3.64E-05 | *TBC1D22A* |

* adjusted for 10 principal components, sex, and cohort of origin

^ adjusted for 10 principal components and sex
