# Supplementary material for: A chromosome 5q31.1 locus associates with tuberculin skin test reactivity in HIV-positive individuals from tuberculosis hyper-endemic regions in east Africa
Source: PLoS Genet. 2017 Jun 19;13(6):e1006710. doi: 10.1371/journal.pgen.1006710 (PMC5495514; doi:10.1371/journal.pgen.1006710)
Supplement: S6 Table — (DOCX) [file pgen.1006710.s006.docx]

**S6 Table.** Association of SNPs with dichotomous tuberculin skin test (at and above 5mm versus below) in the imputed SLC25A48/IL9 region of the combined cohort in a dominant and an additive genetic model; adjusting for 10 principal components, sex, and cohort of origin

| Dominant Model | | | | | | | | | |
| --- | --- | --- | --- | --- | --- | --- | --- | --- | --- |
| SNP | Chr. | Position | Minor Allele | Imputation Certainty | MAF | n | Odds Ratio | 95% Confidence Interval | p value |
| rs17169187 | 5 | 135163758 | C | 0.988 | 0.2389 | 469 | 0.255 | (0.161, 0.403) | 4.57E-09 |
| rs17169180 | 5 | 135161055 | C | 0.993 | 0.2396 | 469 | 0.264 | (0.168, 0.4162) | 9.36E-09 |
| rs13167664 | 5 | 135162467 | G | 0.994 | 0.2396 | 469 | 0.264 | (0.168, 0.4162) | 9.36E-09 |
| rs35520957 | 5 | 135163307 | T | 0.992 | 0.2396 | 469 | 0.264 | (0.168, 0.4162) | 9.36E-09 |
| rs877356 | 5 | 135161418 | T | Not Imputed | 0.2418 | 469 | 0.267 | (0.170, 0.421) | 1.22E-08 |
| Additive Model | | | | | | | | | |
| rs17169187 | 5 | 135163758 | C | 0.988 | 0.2389 | 469 | 0.3196 | (0.214, 0.478) | 2.56E-08 |
| rs17169180 | 5 | 135161055 | C | 0.993 | 0.2396 | 469 | 0.3282 | (0.220, 0.489) | 4.47E-08 |
| rs13167664 | 5 | 135162467 | G | 0.994 | 0.2396 | 469 | 0.3282 | (0.220, 0.489) | 4.47E-08 |
| rs35520957 | 5 | 135163307 | T | 0.992 | 0.2396 | 469 | 0.3282 | (0.220, 0.489) | 4.47E-08 |
| rs877356 | 5 | 135161418 | T | Not Imputed | 0.2418 | 469 | 0.3307 | (0.222, 0.493) | 5.45E-08 |
