# Supplementary material for: A chromosome 5q31.1 locus associates with tuberculin skin test reactivity in HIV-positive individuals from tuberculosis hyper-endemic regions in east Africa
Source: PLoS Genet. 2017 Jun 19;13(6):e1006710. doi: 10.1371/journal.pgen.1006710 (PMC5495514; doi:10.1371/journal.pgen.1006710)
Supplement: S7 Table — (DOCX) [file pgen.1006710.s007.docx]

**S7 Table.** Association of SNPs with continuous tuberculin skin test induration in the imputed *SLC25A48/IL9* region of the combined cohort in a dominant and an additive genetic model; adjusting for 10 principal components, sex, and cohort of origin

| Dominant Model | | | | | | | | | |
| --- | --- | --- | --- | --- | --- | --- | --- | --- | --- |
| SNP | Chr. | Position | Minor Allele | Imputation Certainty | MAF | n | Beta | 95% Confidence Interval | p value |
| rs17169187 | 5 | 135163758 | C | 0.988 | 0.2389 | 469 | -4.285 | (-5.691, -2.88) | 4.58E-09 |
| rs17169180 | 5 | 135161055 | C | 0.993 | 0.2396 | 469 | -4.156 | (-5.564, -2.748) | 1.35E-08 |
| rs13167664 | 5 | 135162467 | G | 0.994 | 0.2396 | 469 | -4.156 | (-5.564, -2.748) | 1.35E-08 |
| rs35520957 | 5 | 135163307 | T | 0.992 | 0.2396 | 469 | -4.156 | (-5.564, -2.748) | 1.35E-08 |
| rs877356 | 5 | 135161418 | T | Not Imputed | 0.2418 | 469 | -4.144 | (-5.552, -2.737) | 1.45E-08 |
| Additive Model | | | | | | | | | |
| rs17169187 | 5 | 135163758 | C | 0.988 | 0.2389 | 469 | -3.430 | (-4.620, -2.240) | 2.84E-08 |
| rs17169180 | 5 | 135161055 | C | 0.993 | 0.2396 | 469 | -3.341 | (-4.534, -2.149) | 6.65E-08 |
| rs13167664 | 5 | 135162467 | G | 0.994 | 0.2396 | 469 | -3.341 | (-4.534, -2.149) | 6.65E-08 |
| rs35520957 | 5 | 135163307 | T | 0.992 | 0.2396 | 469 | -3.341 | (-4.534, -2.149) | 6.65E-08 |
| rs877356 | 5 | 135161418 | T | Not Imputed | 0.2418 | 469 | -3.336 | (-4.529, -2.144) | 6.95E-08 |
