# Supplementary material for: A chromosome 5q31.1 locus associates with tuberculin skin test reactivity in HIV-positive individuals from tuberculosis hyper-endemic regions in east Africa
Source: PLoS Genet. 2017 Jun 19;13(6):e1006710. doi: 10.1371/journal.pgen.1006710 (PMC5495514; doi:10.1371/journal.pgen.1006710)
Supplement: S8 Table — (DOCX) [file pgen.1006710.s008.docx]

**S8 Table.** Most significant SNPs associating with dichotomous TST status in the subset of subjects for whom CD4 counts were available adjusting for 10 principal components, gender, and cohort; and adjusting for 10 principal components, gender, cohort, and CD4 count

| Adjusted for 10 PCs, gender and cohort | | | | | | |
| --- | --- | --- | --- | --- | --- | --- |
| CHR | SNP | BP | A1 | TEST | OR | P |
| 5 | rs877356 | 135161418 | T | DOM | 0.301 | 4.70E-06 |
| 7 | rs7808481 | 10297082 | A | DOM | 2.997 | 1.65E-05 |
| 6 | rs17062122 | 133447417 | C | DOM | 0.343 | 1.67E-05 |
| 9 | rs10817999 | 119903506 | T | DOM | 3.024 | 2.21E-05 |
| 3 | rs9864764 | 190714587 | C | DOM | 0.341 | 2.40E-05 |
| 5 | rs1508801 | 9568765 | C | DOM | 0.260 | 2.46E-05 |
| 4 | rs11944163 | 151204693 | T | DOM | 2.863 | 3.54E-05 |
| 10 | rs12781609 | 134748331 | T | DOM | 0.358 | 3.98E-05 |
| 6 | rs16873911 | 13263140 | G | DOM | 2.943 | 4.36E-05 |
| Adjusted for 10 PCs, gender, cohort, and CD4 count | | | | | | |
| 5 | rs877356 | 135161418 | T | DOM | 0.295 | 3.92E-06 |
| 6 | rs17062122 | 133447417 | C | DOM | 0.307 | 4.49E-06 |
| 9 | rs10817999 | 119903506 | T | DOM | 3.35 | 6.99E-06 |
| 5 | rs1508801 | 9568765 | C | DOM | 0.252 | 1.80E-05 |
| 10 | rs12781609 | 134748331 | T | DOM | 0.350 | 3.13E-05 |
| 3 | rs9864764 | 190714587 | C | DOM | 0.346 | 3.47E-05 |
| 7 | rs7808481 | 10297082 | A | DOM | 2.870 | 4.08E-05 |
| 10 | rs2497338 | 94538960 | G | DOM | 2.786 | 4.73E-05 |
| 6 | rs11967900 | 152022802 | C | DOM | 2.975 | 4.82E-05 |
| 1 | rs12409348 | 2779043 | T | DOM | 3.107 | 4.83E-05 |
