# Supplementary material for: A chromosome 5q31.1 locus associates with tuberculin skin test reactivity in HIV-positive individuals from tuberculosis hyper-endemic regions in east Africa
Source: PLoS Genet. 2017 Jun 19;13(6):e1006710. doi: 10.1371/journal.pgen.1006710 (PMC5495514; doi:10.1371/journal.pgen.1006710)
Supplement: S9 Table — (DOCX) [file pgen.1006710.s009.docx]

**S9 Table.** Interferon gamma release assay results by TST case/control status in the Tanzanian cohort and the Ugandan cohort

| Tanzanian Cohort | | | | | |
| --- | --- | --- | --- | --- | --- |
| IGRA Antigen | TST^-^ | TST^-^ | TST^+^ | TST^+^ | p value ^a^ |
|  | Mean (SD)~~-~~ | Median (IQR) | Mean (SD) | Median (IQR) |  |
| n | 161 |  | 79 |  |  |
| PHA | 18708.11 (27709.84  ) | 8344.84 (2622.43-24400) | 22263.52 (35855.16) | 7233.2 (1699.48-23600) | 0.3988 |
| MEDIA | 184.85 (412.16) | 75 (75-100) | 227.66 (522.57) | 112.5 (75-163.02) | 0.4905 |
| ESAT-6 | 444.77 (1598.35) | 75 (75-194.41) | 4027.45 (9705.44) | 555.59 (140-2305.67) | 8.60E-06 |
| Ag85 | 335.01 (1273.74) | 75 (75-170) | 1387.03 (2772.05) | 374.87 (140-1294.31) | 0.0001 |
| MVS | 220.40 (416.09) | 75 (75-150) | 525.32 (1494.13) | 140 (75-298.90) | 0.0167 |
| WCL | 1366.25 (3454.75) | 230.25 (75-1020.45) | 7711.33 (14517.40) | 3769.24 (1296.55-6832.41) | 3.21E-07 |
| Ugandan Cohort | | | | | |
| MEDIA | 45.27 (75.76) | 1 (1-33.11) | 43.07 (116.80) | 10.07 (1-41.74) | 0.9293 |
| ESAT-6 | 30.14 (34.32) | 4.84 (1-37.81) | 143.73 (498.86) | 23.85 (1-116.18) | 0.3014 |
| CXFT | 101.91 (205.62) | 10.98 (1-69.56) | 1495.00  (422.05) | 407.61 (108.95-970.38) | 0.1757 |
| CFP10 | 69.62 (123.05) | 1 (1-38.84) | 190.69 (713.37) | 29.29 (1-131.79) | 0.4115 |

^a^  Determined by the nonparametric Wilcoxon test.
