# Supplementary material for: A chromosome 5q31.1 locus associates with tuberculin skin test reactivity in HIV-positive individuals from tuberculosis hyper-endemic regions in east Africa
Source: PLoS Genet. 2017 Jun 19;13(6):e1006710. doi: 10.1371/journal.pgen.1006710 (PMC5495514; doi:10.1371/journal.pgen.1006710)
Supplement: S10 Table — (DOCX) [file pgen.1006710.s010.docx]

**S10 Table.** Tuberculin skin test reactivity data at enrollment from the entire Ugandan household contact study

|  | **HIV+ (N=190)** | **HIV- (N=2286)** |
| --- | --- | --- |
| TST+ (%)* | 118 (62%) | 1624 (71%) |
| TST induration (median, range)^ | 16 [5 – 28] | 15 [5 – 33] |

* P = 0.003

^ P=0.06 (Wilcoxon test)
