# Supplementary material for: A chromosome 5q31.1 locus associates with tuberculin skin test reactivity in HIV-positive individuals from tuberculosis hyper-endemic regions in east Africa
Source: PLoS Genet. 2017 Jun 19;13(6):e1006710. doi: 10.1371/journal.pgen.1006710 (PMC5495514; doi:10.1371/journal.pgen.1006710)
Supplement: S11 Table — (DOCX) [file pgen.1006710.s011.docx]

**S11 Table.** Single nucleotide polymorphisms associating with tuberculin skin test dichotomous status (< versus ≥ 5mm) and continuous tuberculin skin test induration using a dominant genetic model in the combined cohort, below a 5x10^-5^ p value after removing patients with possible false negative TST results; adjusted for 10 principal components, sex, and cohort of origin

| TST Dichotomous Status (5mm threshold) | | | | | | | | |
| --- | --- | --- | --- | --- | --- | --- | --- | --- |
| SNP | CHR | Minor Allele | MAF | n | Odds Ratio | 95% Confidence Interval | p value | Nearest gene |
| rs877356 | 5 | T | 0.2241 | 453 | 0.265 | (0.167, 0.4206) | 1.77E-08 | *SLC25A48/IL9* |
| rs7808481 | 7 | A | 0.2163 | 453 | 2.638 | (1.687, 4.127) | 2.14E-05 | *Loc340268* |
| rs697635 | 12 | T | 0.2439 | 453 | 0.3923 | (0.253, 0.610) | 3.14E-05 | *ANKRD33* |
| rs2389096 | 13 | T | 0.24 | 452 | 2.532 | (1.624, 3.948) | 4.14E-05 | *GPC6* |
| rs1880386 | 10 | A | 0.213 | 453 | 2.519 | (1.616, 3.927) | 4.51E-05 | *GRID1* |
| rs9584956 | 13 | G | 0.2406 | 453 | 2.430 | (1.583, 3.731) | 4.92E-05 | *DOCK9* |
| Continuous TST induration | | | | | | | | |
| rs877356 | 5 | T | 0.2241 | 453 | -4.220 | (-5.659, -2.782) | 1.68E-08 | *SLC25A48/IL9* |
| rs697635 | 12 | T | 0.2439 | 453 | -3.197 | (-4.649, -1.746) | 1.95E-05 | *ANKRD33* |
| rs7239554 | 18 | A | 0.2815 | 453 | -3.194 | (-4.644, -1.743) | 1.97E-05 | *C18orf10* |
| rs7808481 | 7 | A | 0.2163 | 453 | 3.172 | (1.697, 4.647) | 3.04E-05 | *Loc340268* |
| rs9920077 | 15 | A | 0.4614 | 453 | 3.433 | (1.827, 5.039) | 3.37E-05 | *KIAA1024* |
| rs12454816 | 18 | A | 0.2263 | 453 | 3.111 | (1.653, 4.570) | 3.50E-05 | *CDH20* |
| rs2389096 | 13 | T | 0.24 | 452 | 3.122 | (1.649, 4.596) | 3.95E-05 | *GPC6* |
