# Supplementary material for: A chromosome 5q31.1 locus associates with tuberculin skin test reactivity in HIV-positive individuals from tuberculosis hyper-endemic regions in east Africa
Source: PLoS Genet. 2017 Jun 19;13(6):e1006710. doi: 10.1371/journal.pgen.1006710 (PMC5495514; doi:10.1371/journal.pgen.1006710)
Supplement: S12 Table — (DOCX) [file pgen.1006710.s012.docx]

**S12 Table.** Single nucleotide polymorphisms associating with tuberculin skin test dichotomous status (< versus ≥ 5mm) and continuous tuberculin skin test induration using a dominant genetic model in the combined cohort, below a 5x10^-5^ p value after removing patients with possible false positive TST reaction to a childhood BCG vaccine; adjusted for 10 principal components, sex, and cohort of origin

| TST Dichotomous Status (5mm threshold) | | | | | | | | |
| --- | --- | --- | --- | --- | --- | --- | --- | --- |
| SNP | CHR | Minor Allele | MAF | n | Odds Ratio | 95% Confidence Interval | p value | Nearest gene |
| rs877356 | 5 | T | 0.2339 | 449 | 0.274 | (0.172, 0.438) | 5.58E-08 | *SLC25A48/IL9* |
| rs1880386 | 10 | A | 0.216 | 449 | 2.587 | (1.656, 4.04) | 2.93E-05 | *GRID1* |
| rs7239554 | 18 | A | 0.2795 | 449 | 0.398 | (0.258, 0.614) | 3.16E-05 | *C18orf10* |
| Continuous TST induration | | | | | | | | |
| rs877356 | 5 | T | 0.2339 | 449 | -3.864 | (-5.303, -2.425) | 2.25E-07 | *SLC25A48/IL9* |
| rs6733728 | 2 | C | 0.3842 | 449 | -3.227 | (-4.690, -1.764) | 1.91E-05 | *Loc402093* |
| rs12454816 | 18 | A | 0.2261 | 449 | 3.160 | (1.708, 4.613) | 2.46E-05 | *CDH20* |
| rs9345216 | 6 | C | 0.2171 | 449 | 3.114 | (1.652, 4.575) | 3.60E-05 | *Loc100129847* |
| rs7239554 | 18 | A | 0.2795 | 449 | -3.072 | (-4.521, -1.623) | 3.91E-05 | *C18orf10* |
| rs6744638 | 2 | G | 0.4376 | 449 | -3.191 | (-4.697, -1.686) | 3.93E-05 | *Loc402093* |
| rs697635 | 12 | T | 0.2428 | 449 | -3.035 | (-4.485, -1.585) | 4.90E-05 | *ANKRD33* |
