# Supplementary material for: A chromosome 5q31.1 locus associates with tuberculin skin test reactivity in HIV-positive individuals from tuberculosis hyper-endemic regions in east Africa
Source: PLoS Genet. 2017 Jun 19;13(6):e1006710. doi: 10.1371/journal.pgen.1006710 (PMC5495514; doi:10.1371/journal.pgen.1006710)
Supplement: S13 Table — (DOCX) [file pgen.1006710.s013.docx]

**S13 Table.** Single nucleotide polymorphisms associating with tuberculin skin test dichotomous status (< versus ≥ 5mm) and continuous tuberculin skin test induration using a dominant genetic model in the combined cohort, below a 5x10^-5^ p value after removing patients with possible false positive TST reaction to a childhood BCG vaccine and possible false negative TST reactions; adjusted for 10 principal components, sex, and cohort of origin

| TST Dichotomous Status (5mm threshold) | | | | | | | | |
| --- | --- | --- | --- | --- | --- | --- | --- | --- |
| SNP | CHR | Minor Allele | MAF | n | Odds Ratio | 95% Confidence Interval | p value | Nearest gene |
| rs877356 | 5 | T | 0.2286 | 433 | 0.272 | (0.169, 0.437) | 7.44E-08 | *SLC25A48/IL9* |
| rs7239554 | 18 | A | 0.2806 | 433 | 0.3823 | (0.246, 0.594) | 1.87E-05 | *C18orf10* |
| rs1880386 | 10 | A | 0.2159 | 433 | 2.650 | (1.682, 4.176) | 2.67E-05 | *GRID1* |
| rs697635 | 12 | T | 0.2471 | 433 | 0.380 | (0.241, 0.600) | 3.25E-05 | *ANKRD33* |
| rs12473869 | 2 | T | 0.2275 | 433 | 2.638 | (1.666, 4.176) | 3.51E-05 | *Loc100131048* |
| rs7326145 | 13 | A | 0.2506 | 433 | 2.581 | (1.643, 4.054) | 3.84E-05 | *COL4A2* |
| rs492479 | 12 | C | 0.2321 | 433 | 0.390 | (0.249, 0.611) | 4.00E-05 | *KSR2* |
| rs10263964 | 7 | C | 0.3557 | 433 | 0.396 | (0.253, 0.619) | 4.77E-05 | *CNTNAP2* |
| Continuous TST induration | | | | | | | | |
| rs877356 | 5 | T | 0.2339 | 433 | -3.951 | (-5.425, -2.477) | 2.39E-07 | *SLC25A48/IL9* |
| rs12454816 | 18 | A | 0.2252 | 433 | 3.397 | (1.914, 4.88) | 9.27E-06 | *CDH20* |
| rs697635 | 12 | T | 0.2471 | 433 | -3.306 | (-4.787, -1.826) | 1.52E-05 | *ANKRD33* |
| rs9345216 | 6 | C | 0.2519 | 433 | 3.277 | (1.783, 4.772) | 2.14E-05 | *Loc100129847* |
| rs7239554 | 18 | A | 0.2806 | 433 | -3.245 | (-4.724, -1.765) | 2.14E-05 | *C18orf10* |
| rs6733728 | 2 | C | 0.3788 | 433 | -3.163 | (-4.656, -1.67) | 3.99E-05 | *Loc402093* |
| rs9920077 | 15 | A | 0.4619 | 433 | 3.439 | (1.805, 5.074) | 4.49E-05 | *KIAA1024* |
| rs10263964 | 7 | C | 0.3557 | 433 | -3.119 | (-4.606, -1.632) | 4.75E-05 | *CNTNAP2* |
