# Supplementary material for: A chromosome 5q31.1 locus associates with tuberculin skin test reactivity in HIV-positive individuals from tuberculosis hyper-endemic regions in east Africa
Source: PLoS Genet. 2017 Jun 19;13(6):e1006710. doi: 10.1371/journal.pgen.1006710 (PMC5495514; doi:10.1371/journal.pgen.1006710)
Supplement: S14 Table — (DOCX) [file pgen.1006710.s014.docx]

**S14 Table.** Association of SNPs with dichotomous tuberculin skin test status (< versus ≥ 5mm) and continuous tuberculin skin test induration in the combined cohort using a dominant genetic model; adjusting for 10 principal components, sex, cohort of origin, and missing IGRA

| TST Dichotomous Status (5mm threshold) | | | | | | | | |
| --- | --- | --- | --- | --- | --- | --- | --- | --- |
| SNP | Chr. | Minor Allele | MAF | n | Odds Ratio | 95% Confidence Interval | p value | Nearest gene |
| rs877356 | 5 | T | 0.2292 | 469 | 0.271 | (0.172, 0.426) | 1.78E-08 | *SLC25A48/IL9* |
| rs7808481 | 7 | A | 0.2164 | 469 | 2.546 | (1.641, 3.948) | 3.00E-05 | *Loc340268* |
| rs12781609 | 10 | T | 0.3214 | 469 | 0.415 | (0.272, 0.633) | 4.37E-05 | *C10orf93* |
| rs2389096 | 13 | T | 0.2382 | 468 | 2.476 | (1.602, 3.828) | 4.49E-05 | *GPC6* |
| Continuous TST induration | | | | | | | | |
| rs877356 | 5 | T | 0.2292 | 469 | -4.094 | (-5.501, -2.687) | 2.12E-08 | *SLC25A48/IL9* |
| rs6974557 | 7 | T | 0.2623 | 469 | -3.065 | (-4.473, -1.656) | 2.44E-05 | *Loc100128056* |
| rs2389096 | 13 | T | 0.2382 | 468 | 3.065 | (1.624, 4.506) | 3.68E-05 | *GPC6* |
| rs7239554 | 18 | A | 0.2804 | 469 | -3.003 | (-4.423, -1.582) | 4.08E-05 | *C18orf10* |
