# Supplementary material for: A chromosome 5q31.1 locus associates with tuberculin skin test reactivity in HIV-positive individuals from tuberculosis hyper-endemic regions in east Africa
Source: PLoS Genet. 2017 Jun 19;13(6):e1006710. doi: 10.1371/journal.pgen.1006710 (PMC5495514; doi:10.1371/journal.pgen.1006710)
Supplement: S15 Table — (DOCX) [file pgen.1006710.s015.docx]

**S15 Table.** Association of SNPs with dichotomous tuberculin skin test status (< versus ≥ 5mm) and continuous tuberculin skin test induration using a dominant genetic model in the combined cohort, including patients with prior TB; adjusting for 10 principal components, sex, and cohort of origin

| TST Dichotomous Status (5mm threshold) | | | | | | | | |
| --- | --- | --- | --- | --- | --- | --- | --- | --- |
| SNP | Chr. | Minor Allele | MAF | n | Odds Ratio | 95% Confidence Interval | p value | Nearest gene |
| rs877356 | 5 | T | 0.2309 | 481 | 0.2725 | (0.174, 0.428) | 1.56E-08 | *SLC25A48/IL9* |
| rs12781609 | 10 | T | 0.3243 | 481 | 0.4166 | (0.275, 0.632) | 3.82E-05 | *C10orf93* |
| rs7808481 | 7 | A | 0.2149 | 481 | 2.472 | (1.605, 3.809) | 4.06E-05 | *Loc340268* |
| Continuous TST induration | | | | | | | | |
| rs877356 | 5 | T | 0.2309 | 481 | -4.053 | (-5.437, -2.669) | 1.70E-08 | *SLC25A48/IL9* |
| rs6733728 | 2 | C | 0.3835 | 481 | -2.986 | (-4.401, -1.571) | 4.19E-05 | *Loc402093* |
| rs16827624 | 2 | A | 0.2359 | 481 | -2.918 | (-4.312, -1.523) | 4.84E-05 | *Loc100131051* |
| rs10051419 | 5 | C | 0.4909 | 480 | 3.410 | (1.780, 5.040) | 4.86E-05 | *OR7H2P* |
