# Supplementary material for: A chromosome 5q31.1 locus associates with tuberculin skin test reactivity in HIV-positive individuals from tuberculosis hyper-endemic regions in east Africa
Source: PLoS Genet. 2017 Jun 19;13(6):e1006710. doi: 10.1371/journal.pgen.1006710 (PMC5495514; doi:10.1371/journal.pgen.1006710)
Supplement: S16 Table — (DOCX) [file pgen.1006710.s016.docx]

**S16 Table.** Association of the rs877356-rs2069885 haplotype using additive genetic models for both SNPs with TST dichotomous status in the *SLC25A48/IL9* region in the combined cohort, the Ugandan cohort and the Tanzanian cohort

| Combined Cohort^1^ | | |
| --- | --- | --- |
| Haplotype | TST^+^-Freq | TST^-^-Freq |
| C-A | 0.0888 | 0.0380 |
| C-G | 0.7404 | 0.6687 |
| T-A | 0.0141 | 0.0264 |
| T-G | 0.1567 | 0.2669 |
| Ugandan Cohort^2^ | | |
| C-A | 0.0867 | 0.0331 |
| C-G | 0.7200 | 0.6097 |
| T-A | 0.0066 | 0.0383 |
| T-G | 0.1867 | 0.3188 |
| Tanzanian Cohort^3^ | | |
| C-A | 0.0968 | 0.0393 |
| C-G | 0.7404 | 0.6687 |
| T-A | 0.0141 | 0.0264 |
| T-G | 0.1567 | 0.2669 |

^1^ Likelihood ratio chisq = 42.90 df = 3 p-value = 2.59E-09*

^2^ Likelihood ratio chisq = 25.84 df = 3 p-value = 1.03E-05^

^3^ Likelihood ratio chisq = 22.06 df = 3 p-value = 6.35E-05^

* adjusted for 10 principal components, sex, and cohort of origin

^ adjusted for 10 principal components and sex
