# Supplementary material for: A chromosome 5q31.1 locus associates with tuberculin skin test reactivity in HIV-positive individuals from tuberculosis hyper-endemic regions in east Africa
Source: PLoS Genet. 2017 Jun 19;13(6):e1006710. doi: 10.1371/journal.pgen.1006710 (PMC5495514; doi:10.1371/journal.pgen.1006710)
Supplement: S17 Table — (DOCX) [file pgen.1006710.s017.docx]

**S17 Table.** Analyses of available and imputed variants using continuous TST scores in the *SLC6A3* region, chromosome 5p15.33 and binary TST scores ^1^, most significant SNPs chromosome 11p14 ^1^ , chromosome 2q14 ^2^, chromosome 2q21-q24 ^2^, chromosome 5p13-q22 ^2^, and chromosome 1q32.1 near *IL-10* ^3^. Only the most significant SNPs are shown.

A)

| *SLC6A3* region, chromosome 5p15.33 and binary TST scores | | | | | | | | | | | |
| --- | --- | --- | --- | --- | --- | --- | --- | --- | --- | --- | --- |
| SNP | Source | Position | Minor Allele | MAF | | Beta | 95% Confidence Interval | | p value | | |
| rs10056116 | Exome Beadchip | 1676638 | A | 0.1779 | | 1.889 | (0.6268, 3.15) | | 0.00351 | | |
| rs13186183 | Imputed | 1791180 | T | 0.0961 | | -2.895 | (-4.56, -1.23) | | 0.00071 | | |
| rs7715002 | Imputed | 1499057 | T | 0.0667 | | 3.361 | (1.369, 5.354) | | 0.00102 | | |
| rs58371035 | Imputed | 1505771 | A | 0.0617 | | 3.390 | (1.318, 5.462) | | 0.00144 | | |
| rs186894420 | Imputed | 1734126 | C | 0.0151 | | 5.972 | (2.243, 9.7) | | 0.0018 | | |
| Most significant SNPs chromosome 11p14 | | | | | | | | | | | |
| rs17234274 | Exome Beadchip | 23214366 | C | 0.3689 | | 0.5821 | (0.4346, 0.7797) | | 2.85E-04 | | |
| rs10834029 | Imputed | 23306545 | G | 0.4973 | | 1.804 | (1.338, 2.431) | | 1.07E-04 | | |
| rs76936560 | Imputed | 23242837 | A | 0.3879 | | 0.5508 | (0.4042, 0.7504) | | 1.57E-04 | | |
| rs1384479 | Imputed | 23243454 | T | 0.3698 | | 0.5541 | (0.4065, 0.7554) | | 1.88E-04 | | |
| rs2449427 | Imputed | 23215101 | C | 0.3852 | | 0.5694 | (0.4205, 0.771) | | 2.71E-04 | | |
| rs17234274 | Imputed | 23214366 | C | 0.3689 | | 0.5723 | (0.423, 0.7741) | | 2.93E-04 | | |
| Chromosome 2q14 | | | | | | | | | | |  |
| rs4848637 | Exome Beadchip | 121579245 | A | 0.1553 | 0.52 | | | (0.3499, 0.775) | | 0.0013 |  |
| rs4848638 | Imputed | 121579604 | A | 0.1555 | 0.5111 | | | (0.3403, 0.7676) | | 0.00122 |  |
| rs12612236 | Imputed | 121459243 | A | 0.3246 | 0.6021 | | | (0.4387, 0.8263) | | 0.00168 |  |
| rs199672409 | Imputed | 121522007 | CT | 0.1817 | 1.881 | | | (1.265, 2.799) | | 0.00181 |  |
| rs12996197 | Imputed | 121464285 | A | 0.3273 | 0.5961 | | | (0.4295, 0.8273) | | 0.00198 |  |
| Chromosome 2q21-q24 | | | | | | | | | | |  |
| rs2521933 | Exome Beadchip | 130457465 | T | 0.2025 | 2.02 | | | (1.414, 2.887) | | 1.13E-04 |  |
| rs2521927 | Imputed | 130456688 | A | 0.1953 | 2.032 | | | (1.418, 2.913) | | 1.12E-04 |  |
| rs2704538 | Imputed | 130403009 | T | 0.2016 | 0.5005 | | | (0.3389, 0.7392) | | 5.03E-04 |  |
| rs2521927 | Imputed | 130404123 | T | 0.1953 | 0.5066 | | | (0.3426, 0.7491) | | 6.56E-04 |  |
| rs2704538 | Imputed | 130401545 | T | 0.2016 | 0.5114 | | | (0.3464, 0.7549) | | 7.38E-04 |  |
| Chromosome 5p13-q22 | | | | | | | | | | |  |
| rs13156567 | Exome Beadchip | 57122376 | A | 0.114 | 2.368 | | | (1.501, 3.737) | | 2.12E-04 |  |
| rs72751331 | Imputed | 57125178 | T | 0.113 | 2.442 | | | (1.527, 3.906) | | 1.95E-04 |  |
| rs10487616 | Imputed | 57105894 | G | 0.1013 | 2.478 | | | (1.531, 4.011) | | 2.21E-04 |  |
| rs1968422 | Imputed | 57138990 | C | 0.3065 | 1.798 | | | (1.314, 2.461) | | 2.44E-04 |  |
| rs13169816 | Imputed | 57145564 | T | 0.1067 | 2.456 | | | (1.501, 4.017) | | 3.46E-04 |  |
| Chromosome 1q32.1 near IL-10 | | | | | | | | | | |  |
| rs6692511 | Exome Beadchip | 206927516 | T | 0.1906 | 1.2 | | | (0.782, 1.843) | | 0.4035 |  |
| rs1800871 | Exome Beadchip | 206946634 | T | 0.3971 | 0.8556 | | | (0.553, 1.317) | | 0.471 |  |
| rs1800896 | Exome Beadchip | 206946897 | G | 0.351 | 1.436 | | | (0.946, 2.381) | | 0.08756 |  |
| rs885334 | Exome Beadchip | 206962416 | A | 0.3877 | 0.9135 | | | (0.603, 1.384) | | 0.6697 |  |

1. Cobat, A., Gallant, C.J., Simkin, L., Black, G.F., Stanley, K., Hughes, J., Doherty, T.M., Hanekom, W.A., Eley, B., Jais, J.P., et al. (2009). Two loci control tuberculin skin test reactivity in an area hyperendemic for tuberculosis. J Exp Med 206, 2583-2591.

2. Stein, C.M., Zalwango, S., Malone, L.L., Won, S., Mayanja-Kizza, H., Mugerwa, R.D., Leontiev, D.V., Thompson, C.L., Cartier, K.C., Elston, R.C., et al. (2008). Genome scan of M. tuberculosis infection and disease in Ugandans. PloS one 3, e4094.

3. Thye, T., Browne, E.N., Chinbuah, M.A., Gyapong, J., Osei, I., Owusu-Dabo, E., Brattig, N.W., Niemann, S., Rusch-Gerdes, S., Horstmann, R.D., et al. (2009). IL10 haplotype associated with tuberculin skin test response but not with pulmonary TB. PLoS One 4, e5420.
