# Supplementary material for: A chromosome 5q31.1 locus associates with tuberculin skin test reactivity in HIV-positive individuals from tuberculosis hyper-endemic regions in east Africa
Source: PLoS Genet. 2017 Jun 19;13(6):e1006710. doi: 10.1371/journal.pgen.1006710 (PMC5495514; doi:10.1371/journal.pgen.1006710)
Supplement: S18 Table — (DOCX) [file pgen.1006710.s018.docx]

**S18 Table.** Single nucleotide polymorphisms associating with TST status near *GAS2* as previously linked to 11p14-15 in Cobat et al ^1^ in the combined cohort, adjusted for 10 principal components, sex, and cohort of origin

| SNP | Position | Minor Allele | Odds Ratio | 95% Confidence Interval | p value |
| --- | --- | --- | --- | --- | --- |
| rs141215155 | 22716709 | C | 0.3183 | (0.1405, 0.7214) | 0.0061 |
| rs147337656 | 22717086 | A | 0.3183 | (0.1405, 0.7214) | 0.0061 |
| rs140137885 | 22722819 | G | 0.3183 | (0.1405, 0.7214) | 0.0061 |
| rs143310525 | 22724201 | C | 0.3183 | (0.1405, 0.7214) | 0.0061 |
| rs147250499 | 22751877 | T | 0.3183 | (0.1405, 0.7214) | 0.0061 |
| rs149974430 | 22711780 | A | 0.3463 | (0.1551, 0.7734) | 0.00969 |
| rs146563332 | 22711993 | C | 0.3463 | (0.1551, 0.7734) | 0.00969 |
| rs113171697 | 22712670 | A | 0.5226 | (0.3126, 0.8736) | 0.0133 |
| rs148539033 | 22822106 | T | 0.3749 | (0.1722, 0.8163) | 0.01346 |

1. Cobat, A., Poirier, C., Hoal, E., Boland-Auge, A., de La Rocque, F., Corrard, F., Grange, G., Migaud, M., Bustamante, J., Boisson-Dupuis, S., et al. (2015). Tuberculin skin test negativity is under tight genetic control of chromosomal region 11p14-15 in settings with different tuberculosis endemicities. J Infect Dis 211, 317-321.
