# Supplementary material for: A chromosome 5q31.1 locus associates with tuberculin skin test reactivity in HIV-positive individuals from tuberculosis hyper-endemic regions in east Africa
Source: PLoS Genet. 2017 Jun 19;13(6):e1006710. doi: 10.1371/journal.pgen.1006710 (PMC5495514; doi:10.1371/journal.pgen.1006710)
Supplement: S19 Table — (DOCX) [file pgen.1006710.s019.docx]

**S19 Table.** Power calculation using a log additive model, a per allele risk of 2.0, a baseline risk of 0.33, an alpha of 0.05, and power of 0.8 in Uganda with a 3 to 1 case to control ratio and Tanzania with a 1 to 2 case to control ratio

| Uganda | |
| --- | --- |
| Minor allele frequency | N TST^+^ needed |
| 0.05 | 699 |
| 0.10 | 374 |
| 0.15 | 267 |
| 0.20 | 215 |
| 0.25 | 185 |
| Tanzania | |
| 0.05 | 240 |
| 0.10 | 130 |
| 0.15 | 94 |
| 0.20 | 76 |
| 0.25 | 66 |
