# Supplementary material for: A chromosome 5q31.1 locus associates with tuberculin skin test reactivity in HIV-positive individuals from tuberculosis hyper-endemic regions in east Africa
Source: PLoS Genet. 2017 Jun 19;13(6):e1006710. doi: 10.1371/journal.pgen.1006710 (PMC5495514; doi:10.1371/journal.pgen.1006710)
Supplement: S20 Table — (DOCX) [file pgen.1006710.s020.docx]

**S20 Table.** Sample sizes for analyses accounting for anergy (variation in IFN-γ response).

|  | DarDar Vaccine Trial extended follow-up | Household Contact Study, Human Exome BeadChip |
| --- | --- | --- |
| Sample size in final analyses | 270 (94 cases / 176 controls) | 199 (150 cases / 49 controls) |
| Removing patients w/ 0mm TST but high IFN ELISA | 257 (94 cases / 163 controls) | 196 (150 cases / 46 controls) |
| Removing patients w/ >5mm TST but low IFN ELISA | 254 (78 cases / 176 controls) | 195 (146 cases / 49 controls) |
| Removing patients w/ missing IFN ELISA | 237 (84 cases / 153 controls) | 97 (79 cases / 18 controls) |
