# Supplementary material for: A chromosome 5q31.1 locus associates with tuberculin skin test reactivity in HIV-positive individuals from tuberculosis hyper-endemic regions in east Africa
Source: PLoS Genet. 2017 Jun 19;13(6):e1006710. doi: 10.1371/journal.pgen.1006710 (PMC5495514; doi:10.1371/journal.pgen.1006710)
Supplement: S21 Table — (DOCX) [file pgen.1006710.s021.docx]

**S21 Table.** Exclusions following Quality Control.

| Exclusion Criteria | DarDar Vaccine Trial extended follow-up | Household Contact Study, Human Exome BeadChip |
| --- | --- | --- |
| Original sample size | 304 | 263 |
| Passed pre-genotyping DNA quality check | 301 | 263 |
| No reported prior TB disease | 283 | 261 |
| Age > 18 | 283 | 234 |
| Relatedness < 0.2 pi hat | 274 | 213 |
| Per individual genotyping efficiency > 95% | 274 | 213 |
| Available TST results with PHA reactive T cells | 270 (94 cases / 176 controls) | 199 (150 cases / 49 controls) |
| Final sample size for analyses | 270 (94 cases / 176 controls) | 199 (150 cases / 49 controls) |
