# Supplementary material for: A chromosome 5q31.1 locus associates with tuberculin skin test reactivity in HIV-positive individuals from tuberculosis hyper-endemic regions in east Africa
Source: PLoS Genet. 2017 Jun 19;13(6):e1006710. doi: 10.1371/journal.pgen.1006710 (PMC5495514; doi:10.1371/journal.pgen.1006710)
Supplement: S1 Fig — (DOCX) [file pgen.1006710.s022.docx]

**S1 Figure.** QQ plot of results from a logistic regression association of tuberculin skin test status (< versus ≥ 5mm) with a dominant genetic model of available SNPs for the combined Ugandan and Tanzanian datasets

**
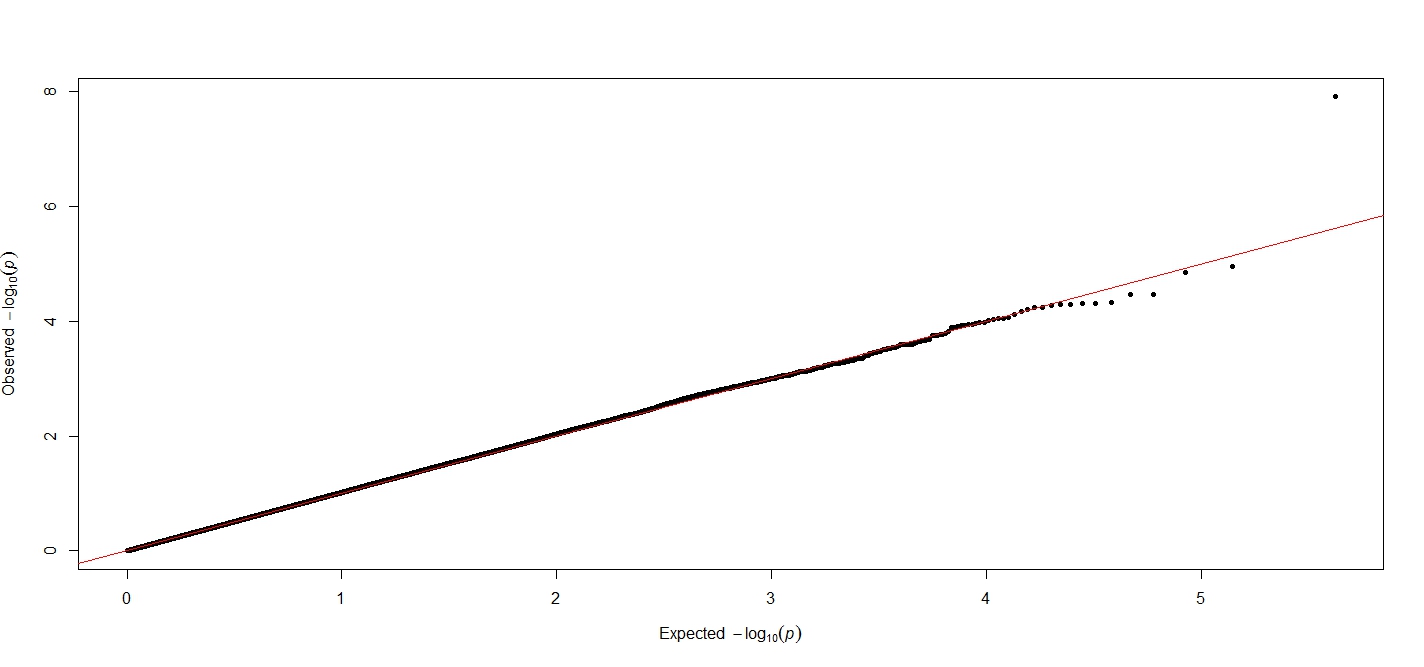
**
