# Supplementary material for: A chromosome 5q31.1 locus associates with tuberculin skin test reactivity in HIV-positive individuals from tuberculosis hyper-endemic regions in east Africa
Source: PLoS Genet. 2017 Jun 19;13(6):e1006710. doi: 10.1371/journal.pgen.1006710 (PMC5495514; doi:10.1371/journal.pgen.1006710)
Supplement: S3 Fig — (DOCX) [file pgen.1006710.s024.docx]

**S3 Figure.** Locus zoom plot of results from a logistic regression of case/control tuberculin skin test induration status (< versus ≥ 5mm) with SNPs in the *SLC25A48/IL9* region using a dominant genetic model in the combined cohort, adjusted for 10 principal components, sex, and cohort of origin

**
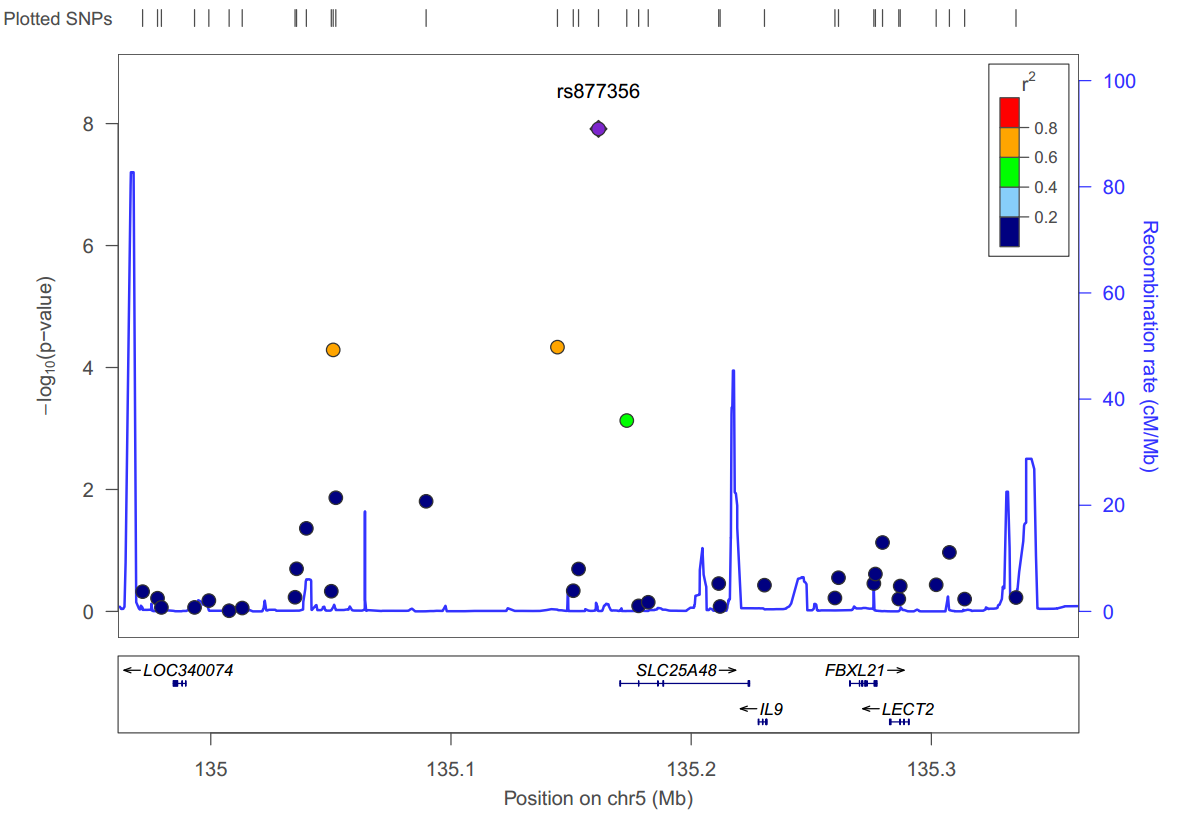
**
