# Supplementary material for: A chromosome 5q31.1 locus associates with tuberculin skin test reactivity in HIV-positive individuals from tuberculosis hyper-endemic regions in east Africa
Source: PLoS Genet. 2017 Jun 19;13(6):e1006710. doi: 10.1371/journal.pgen.1006710 (PMC5495514; doi:10.1371/journal.pgen.1006710)
Supplement: S5 Fig — (DOCX) [file pgen.1006710.s026.docx]

**S5 Figure.** Locus zoom plot of results from a logistic regression association of case/control tuberculin skin test induration status (< versus ≥ 5mm) with a dominant genetic model of SNPs in the *SLC25A48/IL9* region in the Tanzanian cohort, adjusted for 10 principal components and sex

**
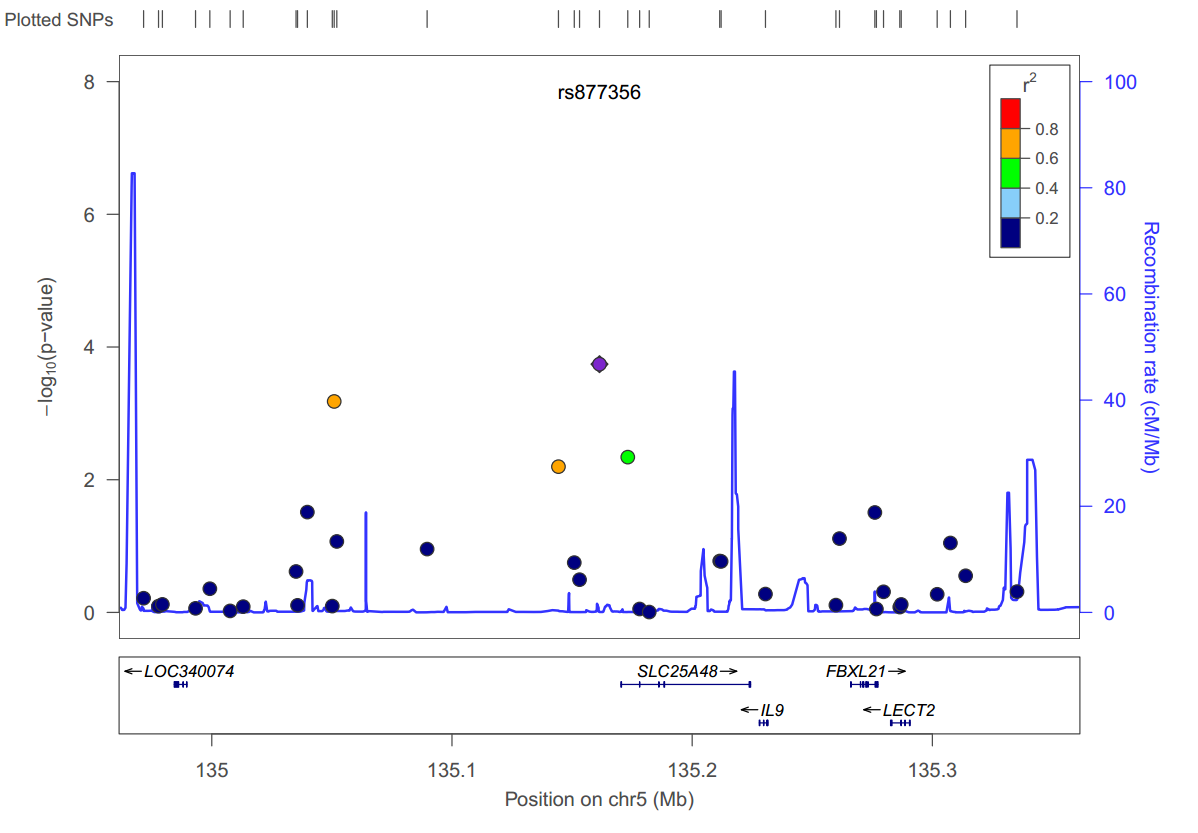
**
