# Supplementary material for: A chromosome 5q31.1 locus associates with tuberculin skin test reactivity in HIV-positive individuals from tuberculosis hyper-endemic regions in east Africa
Source: PLoS Genet. 2017 Jun 19;13(6):e1006710. doi: 10.1371/journal.pgen.1006710 (PMC5495514; doi:10.1371/journal.pgen.1006710)
Supplement: S6 Fig — (DOCX) [file pgen.1006710.s027.docx]

**S6 Figure.** Haploview plots of the rs877356 region using the D’ metric in A) the Tanzanian cohort and B) the Ugandan cohort

A)

**
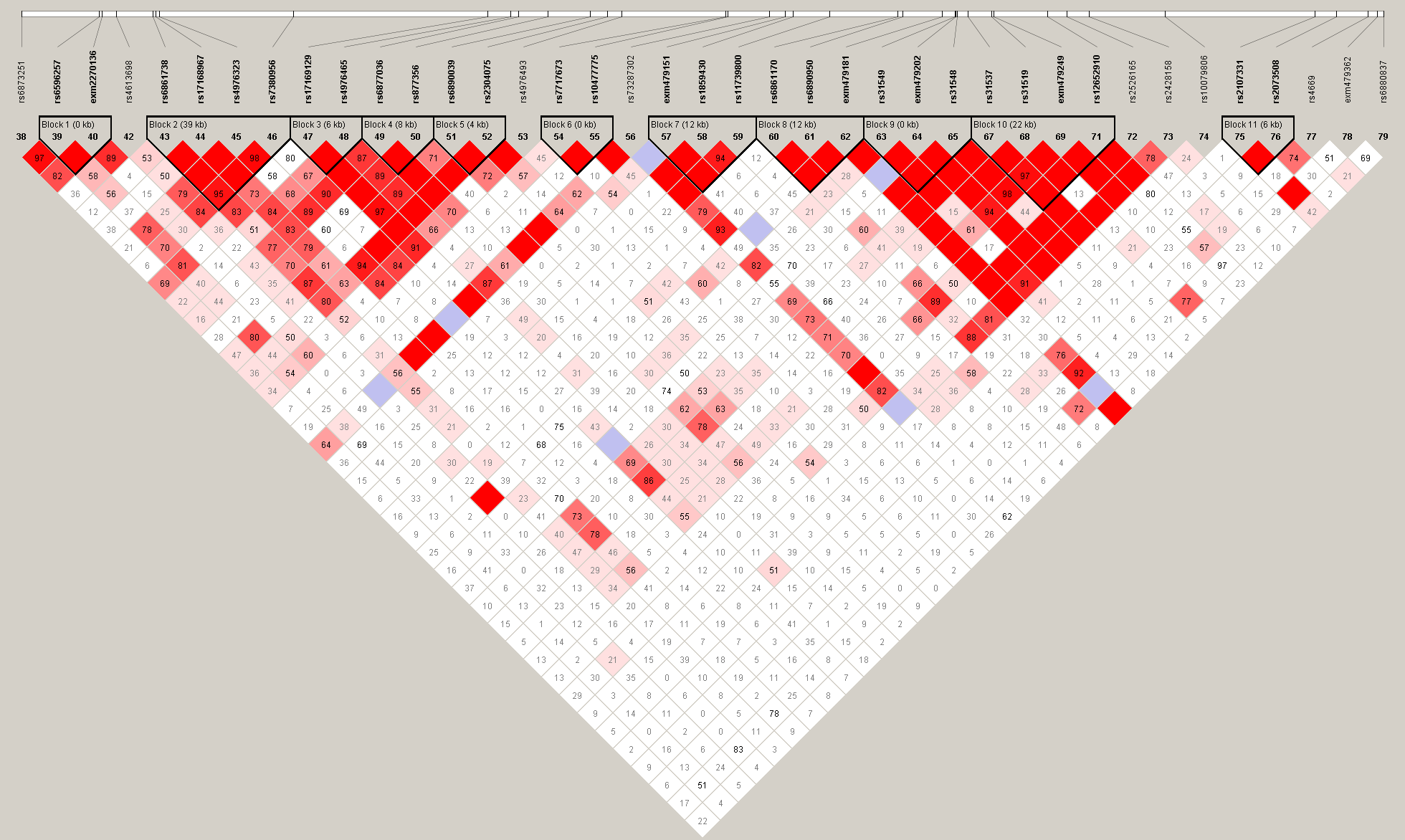
**

B)

**
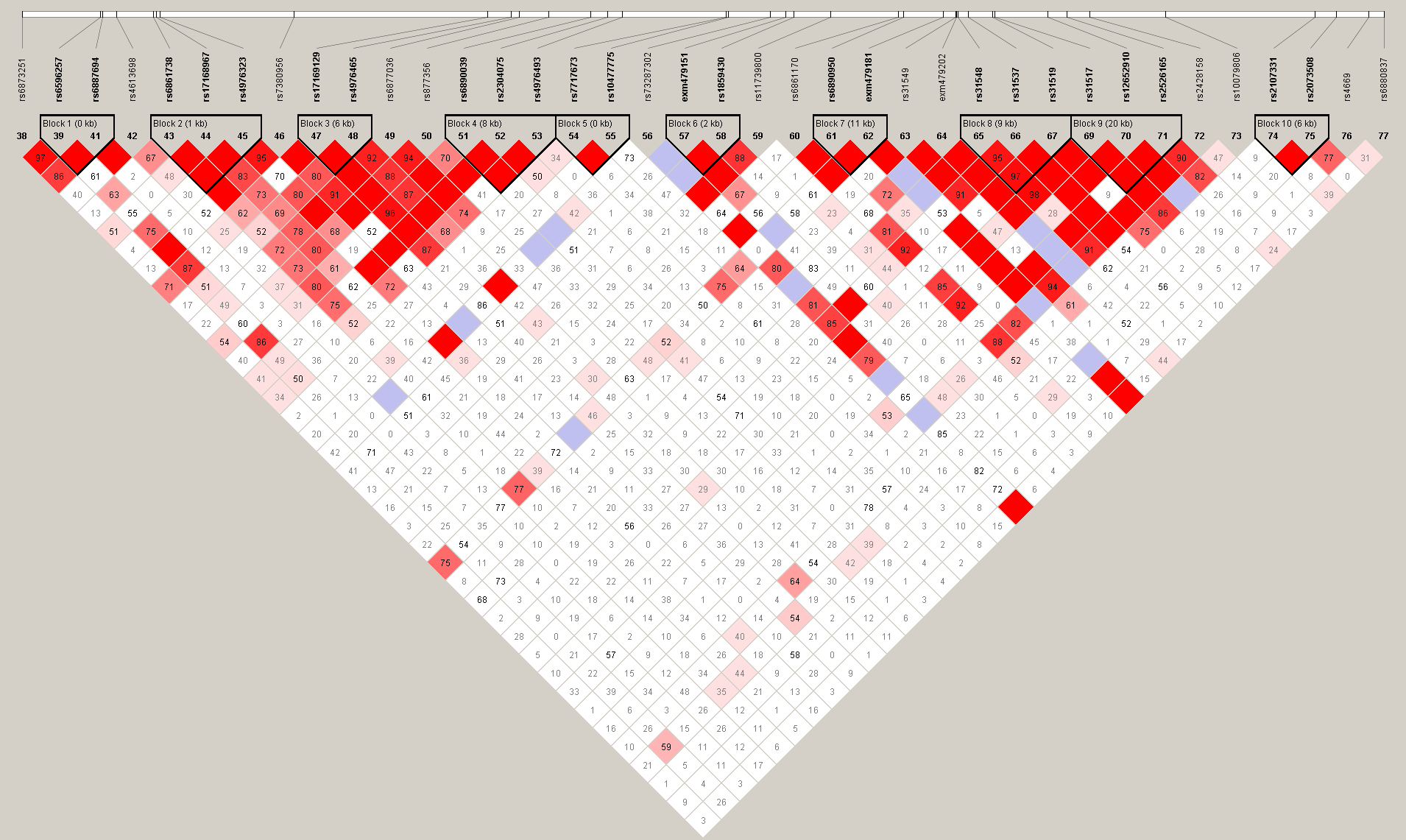
**
