# Supplementary figures and images for: A chromosome 5q31.1 locus associates with tuberculin skin test reactivity in HIV-positive individuals from tuberculosis hyper-endemic regions in east Africa
Source: PLoS Genet. 2017 Jun 19;13(6):e1006710. doi: 10.1371/journal.pgen.1006710 (PMC5495514; doi:10.1371/journal.pgen.1006710)

**S8 Figure.** Cluster plot for rs877356. Cases and controls were run together to minimize batch effects.


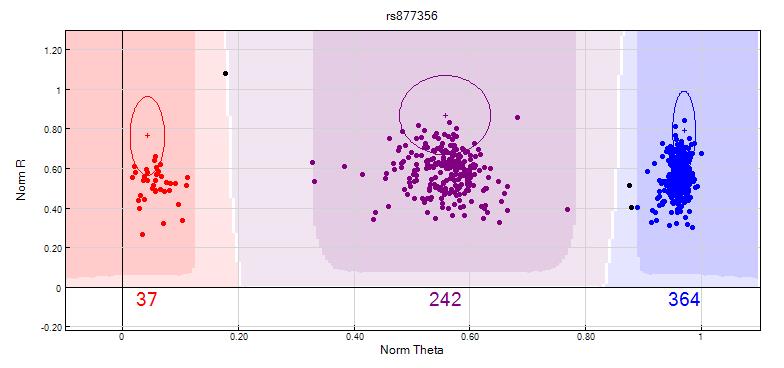

Supplement: S8 Fig — Cases and controls were run together to minimize batch effects. (DOCX) [file pgen.1006710.s029.docx]
